# The serum uric acid-to-high-density lipoprotein cholesterol ratio is a predictor for all-cause and cardiovascular disease mortality: a cross-sectional study

**DOI:** 10.3389/fendo.2024.1417485

**Published:** 2024-09-13

**Authors:** Zhanbin Li, Qiaoran Liu, Zhenyu Yao

**Affiliations:** ^1^ Department of Endocrinology, Shandong Provincial Hospital, Shandong University; Key Laboratory of Endocrine Glucose & Lipids Metabolism and Brain Aging, Ministry of Education, Jinan, Shandong, China; ^2^ Shandong Clinical Research Center of Diabetes and Metabolic Diseases, Jinan, Shandong, China; ^3^ Shandong Institute of Endocrine and Metabolic Diseases, Jinan, Shandong, China; ^4^ “Chuangxin China” Innovation Base of Stem Cell and Gene Therapy for Endocrine Metabolic Diseases, Jinan, Shandong, China; ^5^ Shandong Engineering Laboratory of Prevention and Control for Endocrine and Metabolic Diseases, Jinan, Shandong, China; ^6^ Shandong Engineering Research Center of Stem Cell and Gene Therapy for Endocrine and Metabolic Diseases, Jinan, Shandong, China; ^7^ Department of Breast Surgery, Shandong Provincial Qianfoshan Hospital, Shandong University, Jinan, Shandong, China

**Keywords:** the serum uric acid-to-high-density lipoprotein cholesterol ratio, mortality, cardiovascular disease, obese, diabetes, NHANES

## Abstract

**Objective:**

The exact relationship between the serum uric acid-to-HDL cholesterol ratio (UHR) and mortality rates remains enigmatic among American adults. This study aims to clarify the association between UHR and both all-cause and cardiovascular disease (CVD) mortality in US adults.

**Methods:**

This study enrolled 48054 patients from the National Health and Nutrition Examination Survey (NHANES). Mortality outcomes were determined by linking to National Death Index (NDI) records up to December 31,2019. Multivariate Cox proportional hazards models were constructed to analyze explore the associations between UHR and mortality. Dose-response relationships were explored using restricted cubic splines, and stratified analyses were conducted based on gender, age, race, education, PIR, smoking status, alcohol intake, physical activity, BMI, diabetes and hypertension.

**Results:**

During the follow-up period, the overall mortality for all-cause and CVD was 10.9% and 2.7%, respectively. The adjusted HRs in the highest quintile were 1.16 (95% CI: 1.05, 1.29) for all-cause mortality and 1.2 (95% CI: 1, 1.45) for CVD mortality. In diabetes, obese, and CVD subgroups, significantly elevated adjusted HRs were observed for both all-cause and CVD mortality. Specifically, diabetes patients had adjusted HRs of 1.32 (95% CI: 1.11, 1.57) and 1.38 (95% CI: 1.01, 1.90), obese individuals had HRs of 1.32 (95% CI: 1.10, 1.58) and 1.55 (95% CI: 1.06, 2.28), and CVD patients had HRs of 1.29 (95% CI: 1.10, 1.50) and 1.38 (95% CI: 1.06, 1.79), respectively. A non-linear relationship between UHR and mortality was identified, with critical thresholds of 12.4 for all-cause mortality and 10.7 for CVD mortality in the general population. Significant interactions were observed between UHR and stratified variables, including gender, BMI, education, smoking, alcohol use, and hypertension for all-cause mortality, while significant interactions were observed based on gender, smoking, and alcohol intake for CVD mortality. Comparable trends were also observed in patient with diabetes, obese and CVD.

**Conclusions:**

In this cohort study, we provide novel insights into the association between serum UHR concentrations and mortality in the general population. UHR is a strong predictor of all-cause and cardiovascular mortality in the general population.

## Background

Cardiovascular disease (CVD), the foremost cause of mortality globally, remains a formidable barrier to public health (1, 2). Despite remarkable treatment breakthroughs, patients with CVD still grapple with persistent recurrences (3), maintaining a stubbornly high mortality rate (1). Unraveling the prognostic factors for CVD patients offers a promising avenue to significantly reduce the global mortality burden, particularly cardiovascular mortality. Ideally, prognostic factors should be independently identifiable, cost-effective, and seamlessly integrated into clinical practice for enhanced prognostic precision and patient care.

In clinical practice, serum uric acid (UA) (4–9) and high-density lipoprotein cholesterol (HDL-C) (10–12) have been linked to cardiovascular disease (CVD) and adverse events. However, comorbidities affecting renal excretion and lipid metabolism limit their predictive accuracy (13–15). This underscores the importance of a more comprehensive, multifaceted evaluation. In this context, the serum uric acid-to-HDL-cholesterol ratio (UHR) emerges as a promising marker. Studies show a strong correlation between UHR and CVD, including atherosclerosis (16), ischemic heart disease (17–20), hypertension (21), acute myocardial infarction (22), coronary artery disease (CAD) (23, 24) and acute coronary syndrome (18). More importantly, studies have shown that the UHR predicts the onset of coronary artery disease better than UA or HDL-C alone in patients with chronic kidney disease (23). Additionally, UHR is correlated with CVD risk factors, including insulin resistance (25, 26), visceral fat accumulation (27, 28), and is also associated with metabolic diseases such as diabetes (29–31), metabolic syndrome (32), metabolism dysfunction-associated fatty liver disease (33–36), chronic kidney disease (37), Hashimoto’s thyroiditis (38). UHR’s broad correlation with these factors and conditions underscores its value in CVD risk assessment, integrating several key risk indicators.

Despite the remarkable potential of the UHR in forecasting CVD, investigations into its correlation with adverse cardiovascular outcomes, notably CVD mortality, are still limited. To date, there is only one study that has identified UHR as a predictive factor for cardiovascular mortality in patients undergoing peritoneal dialysis (39). The question of whether UHR can similarly predict cardiovascular mortality in the general population and among specific subgroups remains elusive. Therefore, our study endeavors to bridge this knowledge gap by examining the influence of UHR on mortality and elucidating the dose-response relationship, utilizing data from the National Health and Nutrition Examination Survey (NHANES) across diverse American populations.

## Methods

### Study population and design

NHANES, a comprehensive, multistage survey conducted by the U.S. Centers for Disease Control and Prevention’s National Center for Health Statistics, collects demographic, socioeconomic, dietary, physiological, and laboratory data through interviews and medical exams. NHANES has received ethical approval from the CDC’s research ethics review board [NHANES 1999-2004: Protocol #98-12; NHANES 2005-2010; Protocol #2005-06; NHANES 2011-2018: Protocol #2011-17, #2018-01 (Effective beginning October 26, 2017)7]. NHANES ensures participant rights protection through informed written consent. Datasets from NHANES, including those used in our study, are publicly accessible on the official NHANES website (https://www.cdc.gov/nchs/nhanes/index.html).

Criteria for subgroup division are as follows: diabetes, defined by the American Diabetes Association (ADA), includes self-reported diagnosis, insulin/oral hypoglycemic use, fasting blood glucose ≥ 126 mg/dL, or HbA1c ≥ 6.5% (40). BMI is calculated as weight divided by height squared, with obesity defined as BMI ≥ 30 kg/m² (41), and CVD diagnosis is determined through self-reported physician diagnoses during interviews using a standardized questionnaire on CHF/CHD/angina pectoris/MI/stroke, with affirmative answers indicating the presence of CVD.

**Figure 1:**
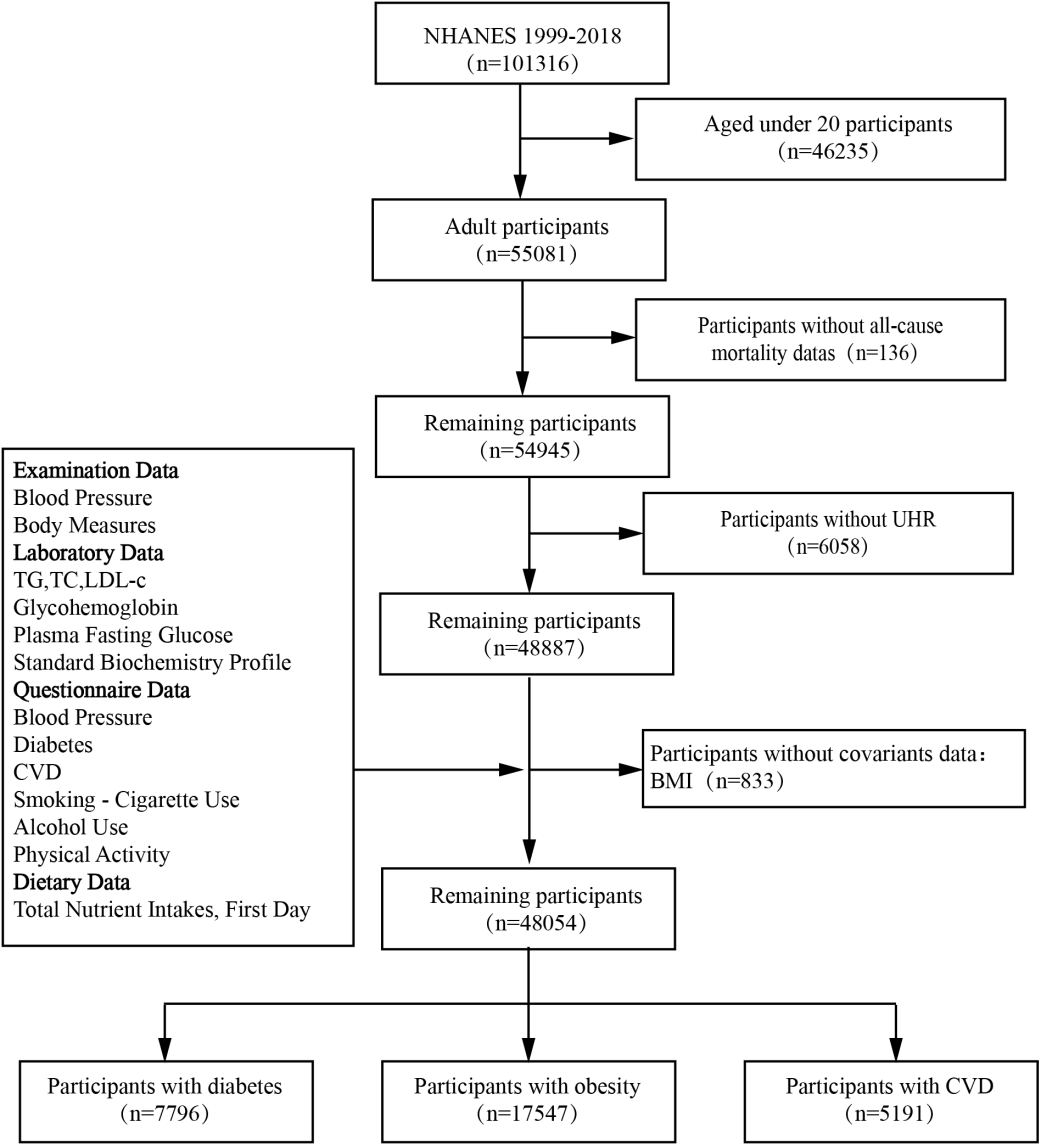
The selection flowchart of the participants.

Patients were excluded if they met any of the following criteria: 1) age < 20 years; 2) missing death status information; 3) incomplete data on UA levels and HDL-C values; or 4) missing covariant data. A final cohort of 48,054 patients from NHANES 1999–2018 was included in the study (**Figure 1**).

### Assessment of UHR

The exposure variable was the UHR, calculated as serum UA divided by HDL- C. UA measurements were performed using various multichannel analyzers across NHANES cycles (Hitachi Model 704, Beckman Synchron LX20, Beckman UniCel DxC800 Synchron, and Roche Cobas 6000). Fasting serum HDL- C concentrations were measured using the ARCHITECT auto-analyzer and Abbott reagent kits. Participants were categorized into four groups (Q1-Q4) based on UHR quartiles, with Q1 serving as the reference group.

### Outcome ascertainment

The primary endpoint was all-cause mortality, with CVD-specific mortality as a secondary outcome. Mortality status was determined using the NHANES Public-Use Linked Mortality File, updated until December 31, 2019 (https://www.cdc.gov/nchs/data-linkage/mortality-public.htm), which linked to the NDI data with a probabilistic matching algorithm to determine mortality status (42). The disease-specific mortality data in the NDI have been identified according to the International Statistical Classification of Diseases, 10th Revision (ICD-10), with only a relatively slight possibility of misclassification. Specific mortality was defined as death due to heart diseases (054–064), malignant neoplasms (019–043), and all other causes (010) for our study (43). Follow-up time for each person was calculated as the difference between the baseline examination date and the last known date alive or censored from the mortality file.

### Assessment of covariates

Confounding factors potentially associated with mortality were enrolled in this analysis. Information on age, sex, race or ethnicity, education level, and family income was collected from the demographic data. Race was categorized as Mexican American, non-Hispanic White, non-Hispanic Black, or other race, while education level was grouped as less than high school, high school or equivalent, or college or above. Family economic status was determined by income to poverty ratio (PIR), with three categories: < 1.30, 1.31 to 3.50, and ≥ 3.50 (44).

Smoking status, alcoholic intake, and physical activity were assessed via standardized questionnaires. Participants were categorized into nonsmokers, former smokers, and current smokers based on smoking history and habits. Alcohol consumption was determined using a 24-hour dietary recall, classifying individuals as nondrinkers, moderate drinkers (0.1-27.9 g/day for men, 0.1-13.9 g/day for women), or heavy drinkers (≥ 28 g/day for men, ≥ 14 g/day for women). Physical activity was divided into inactive, active (meeting recommended levels), and insufficiently active categories based on previous literature (45).

Clinical indicators such as alanine aminotransferase (ALT), aspartate aminotransferase (AST), blood urea nitrogen (BUN), serum creatinine (Scr), gamma-glutamyltransferase(GGT), lactate dehydrogenase (LDH), total bilirubin (Tbil), fasting blood glucose (FBG), glycosylated hemoglobin A1c (HbA1c), triglycerides (TG), total cholesterol (TC), low-density lipoprotein cholesterol (LDL-C), and HDL-C were measured in the NHANES laboratory following the relevant standardized protocols. The estimated glomerular filtration rate (eGFR) was calculated using the Chronic Kidney Disease Epidemiology Collaboration Equation (46).

Hypertension was defined as having a history of hypertension, systolic blood pressure ≥ 130 mmHg, or diastolic blood pressure ≥ 80 mmHg, according to the 2017 American College of Cardiology and American Heart Association hypertension guidelines.

### Statistical analysis

The statistical analysis was performed by using R software (version 4.3.1; https://www.r-project.org). Given the complicated sampling design, NHANES weights and strata variables were considered when calculating statistics (47). Data categorized into continuous (mean ± SD) and categorical (percentages) variables. Statistical analysis of continuous variables employed Student’s t-test or Mann–Whitney U test, depending on data distribution. Categorical variables were compared using chi-square test. Obesity phenotype stratified, baseline characteristics compared using one-way ANOVA.

Study participants were divided into four quartiles (Q1-Q4) of UHR. Baseline characteristics were compared across quartiles using ANOVA and chi-square tests. Incidence rates of all-cause and CVD mortality were computed for each quartile during follow-up.

To evaluate the independent predictive value of the UHR, we developed multivariate weighted Cox proportional hazards regression models with three models to control for confounding factors. Model 1 was unadjusted, Model 2 was adjusted for age, race, and gender, and Model 3 was adjusted for age, gender, race, education level, family income level, BMI, smoking status, alcohol intake, diabetes and hypertension. We utilized the restricted cubic spline (RCS) model to graphically represent the dose-response relationship between UHR levels and both all-cause and CVD mortality.

Stratified analyses were performed in the strata of age (< 30,30-40,40-50 or ≥ 50 years old), sex (male or female), race or ethnicity (White, Black, Mexican, or Other), education level (less than high school, high school or equivalent, or college or above), family income level(<1.30, 1.31 to 3.50, and ≥ 3.50), smoking status (current smoker, former smoker or nonsmoker), alcohol intake (heavy drinking, moderate drinking or nondrinking), physical activity(active, insufficiently or active), BMI (< 25 or 25-30, ≥ 30 kg/m^2^), diabetes and hypertension. A *P*-value of less than 0.05 was considered statistically significant.

## Results

### Baseline characteristics of study participants

**Table 1** outlines the baseline characteristics of 48,054 participants stratified by UHR quartiles. The average age of the participants was 46.99 ± 16.85 years, and 51.8% of them were women. During a mean follow-up of 8.05 ± 5.17 years, the mortality of all-cause, CVD and malignant neoplasms were 10.9%, 2.7% and 2.5%, respectively. Average UHR in the enrolled patients was 5.4 ± 1.42. Participants with higher UHR level tended to be older, male, obese, and had a higher prevalence of comorbidities. They exhibited significantly higher all-cause and CVD mortality rates. Among all subjects, there were 7796 patients with diabetes, 17547 patients with obesity, and 5191 patients with CVD. Detailed characteristics of patients with diabetes, obesity and CVD are provided in **Supplementary Tables 1–3** of the **Supplementary Material**.

**Table 1:** Baseline characteristics of participants.

	Overal	The UHR quartiles^a^				*P*
		≤7.54	7.54-10.43	10.43-14.19	>14.19	
n (cases)	202156431.6	51971562.8	49440178.08	50561106.09	50183584.61	
Gender [Female (%)]	104687265.4 (51.8)	44827046.3 (86.3)	30468895.3 (61.6)	19284634.1 (38.1)	10106689.7 (20.1)	<0.001
Age (years)	46.99 ± 16.85	46.6 ± 16.63	47 ± 17.2	47.38 ± 16.85	47.01 ± 16.73	0.058
Race (%)						<0.001
Mexican American	16644088.2 (8.2)	3648695.5 (7.0)	4225619.2 (8.5)	4453138.1 (8.8)	4316635.5 (8.6)	
Other race	25192744.8 (12.5)	6153615.3 (11.8)	6162769.6 (12.5)	6312241.4 (12.5)	6564118.4 (13.1)	
Non-Hispanic White	138834120.3 (68.7)	36185001.0 (69.6)	33174168.3 (67.1)	34545745.2 (68.3)	34929205.8 (69.6)	
Non-Hispanic Black	21485478.2 (10.6)	5984251.0 (11.5)	5877621.0 (11.9)	5249981.4 (10.4)	4373624.9 (8.7)	
Education (%)						<0.001
Less than high school	34697307.6 (17.2)	7111915.3 (13.7)	8815042.1 (17.8)	9027755.4 (17.9)	9742594.9 (19.4)	
High school	48371833.5 (23.9)	10445913.6 (20.1)	11806768.0 (23.9)	12589035.8 (24.9)	13530116.1 (27.0)	
College or above	118912435.0 (58.8)	34378002.7 (66.1)	28762629.0 (58.2)	28916889.7 (57.2)	26854913.6 (53.5)	
Missing data ^c^	174855.5 (0.1)	35731.3 (0.1)	55739.0 (0.1)	27425.2 (0.1)	55960.0 (0.1)	
PIR (%)						<0.001
<1.30	39612722.5 (19.6)	8877508.5 (17.1)	10163751.9 (20.6)	10237321.0 (20.2)	10334141.1 (20.6)	
1.31-3.50	67367994.0 (33.3)	16429692.4 (31.6)	16536231.6 (33.4)	17063335.8 (33.7)	17338734.2 (34.6)	
≥3.50	80861327.2 (40.0)	22748699.6 (43.8)	19273129.9 (39.0)	19840101.9 (39.2)	18999395.8 (37.9)	
Missing data ^c^	14314387.8 (7.1)	3915662.3 (7.5)	3467064.7 (7.0)	3420347.4 (6.8)	3511313.4 (7.0)	
All-cause mortality (%)	22059155.3 (10.9)	4571034.2 (8.8)	5048167.8 (10.2)	5501256.1 (10.9)	6938697.3 (13.8)	<0.001
CVD mortality (%)	5407837.3 (2.7)	999293.2 (1.9)	1110675.9 (2.2)	1400666.7 (2.8)	1897201.4 (3.8)	<0.001
Malignant neoplasms mortality (%)	5107016.4 (2.5)	1059275.7 (2.0)	1168394.9 (2.4)	1360012.7 (2.7)	1519333.1 (3.0)	<0.001
Time(years)	9.95 ± 5.65	9.95 ± 5.65	9.93 ± 5.66	9.97 ± 5.6	9.93 ± 5.67	0.946
UHR (%)	11.32 ± 5.23	5.82 ± 1.17	8.96 ± 0.83	12.15 ± 1.06	18.5 ± 4.25	<0.001
ALB(g/L)	42.77 ± 3.48	42.41 ± 3.65	42.63 ± 3.47	43 ± 3.42	43.03 ± 3.34	<0.001
ALT (U/L)	25.47 ± 22.57	20.28 ± 13.84	22.81 ± 18.97	26.54 ± 17.61	32.41 ± 33.21	<0.001
AST (U/L)	25.13 ± 16.04	23.45 ± 13.13	23.99 ± 15.93	25.45 ± 16.08	27.64 ± 18.36	<0.001
Tbil (umol/L)	11.53 ± 5.39	10.77 ± 4.9	11.22 ± 5.18	11.89 ± 5.59	12.29 ± 5.73	<0.001
GGT (IU/L)	28.54 ± 41.59	22.09 ± 33.71	25.77 ± 39.67	30.7 ± 49.34	35.78 ± 40.98	<0.001
LDH(IU/L)	133.41 ± 30.74	131.11 ± 29.48	132.86 ± 30.8	133.5 ± 29.8	136.22 ± 32.62	<0.001
BUN (mmol/L)	4.84 ± 1.93	4.41 ± 1.63	4.7 ± 1.71	4.96 ± 1.91	5.28 ± 2.3	<0.001
Scr (umol/L)	77.66 ± 32.71	67.22 ± 25.1	74.49 ± 29.33	80.94 ± 30.8	88.3 ± 40.09	<0.001
eGFR (mL/min/1.73 m2)	101.79 ± 30.86	110.8 ± 34.6	103.59 ± 29.8	98.63 ± 28.03	93.88 ± 27.76	<0.001
TC (mmol/L) ^b^	5.08 ± 1.08	5.16 ± 1	5.06 ± 1.06	5.05 ± 1.11	5.05 ± 1.16	<0.001
TG (mmol/L) ^b^	1.5 ± 1.33	1 ± 0.54	1.24 ± 0.99	1.55 ± 1.08	2.22 ± 1.96	<0.001
LDL-C (mmol/L) ^b^	3 ± 0.92	2.86 ± 0.87	3.01 ± 0.9	3.08 ± 0.95	3.04 ± 0.95	<0.001
BMI (kg/m2)	28.77 ± 6.72	25.49 ± 5.4	28.15 ± 6.33	29.76 ± 6.7	31.79 ± 6.74	<0.001
HbA1c (%)	5.57 ± 0.92	5.4 ± 0.8	5.54 ± 0.89	5.63 ± 0.94	5.72 ± 0.99	<0.001
FPG (mmol/L) ^b^	5.84 ± 1.72	5.47 ± 1.44	5.75 ± 1.65	5.99 ± 1.85	6.16 ± 1.84	<0.001
Diabetes (%)						<0.001
No	178002180.0 (88.1)	48762598.4 (93.8)	44370413.9 (89.7)	43636421.7 (86.3)	41232746.0 (82.2)	
Yes	24154251.6 (11.9)	3208964.4 (6.2)	5069764.2 (10.3)	6924684.4 (13.7)	8950838.7 (17.8)	
Hypertension (%)						<0.001
No	103046265.1 (51.0)	32637494.8 (62.8)	26753863.4 (54.1)	23683920.8 (46.8)	19970986.1 (39.8)	
Yes	99074121.1 (49.0)	19327299.7 (37.2)	22670648.8 (45.9)	26866702.6 (53.1)	30209470.0 (60.2)	
Missing data ^c^	36045.4 (0.0)	6768.3 (0.0)	15666.0 (0.0)	10482.7 (0.0)	3128.5 (0.0)	
Smoking status (%)						<0.001
Current smokers	43201648.6 (21.4)	9264230.9 (17.8)	10637060.8 (21.5)	11508643.9 (22.8)	11791713.0 (23.5)	
Former smokers	50238986.9 (24.9)	11417592.6 (22.0)	11043733.2 (22.3)	13493747.9 (26.7)	14283913.2 (28.5)	
Non-smokers	108606961.8 (53.7)	31262680.8 (60.2)	27749339.5 (56.1)	25517851.2 (50.5)	24077090.4 (48.0)	
Missing data ^c^	108834.1 (0.1)	27058.4 (0.1)	10044.6 (0.0)	40863.1 (0.1)	30868.0 (0.1)	
Alcohol consumption (%)						<0.001
Heavy drinking	35474457.6 (17.5)	10779188.1 (20.7)	9093401.0 (18.4)	8312898.3 (16.4)	7288970.0 (14.5)	
Moderate drinking	17278527.1 (8.5)	3599253.4 (6.9)	4072538.6 (8.2)	4835566.2 (9.6)	4771168.9 (9.5)	
Non-drinkers	139969963.9 (69.2)	34922824.2 (67.2)	33785477.7 (68.3)	35244158.6 (69.7)	36017503.4 (71.8)	
Missing data ^c^	9433483.1 (4.7)	2670297.1 (5.1)	2488760.8 (5.0)	2168482.9 (4.3)	2105942.2 (4.2)	
Physical activity (%)						<0.001
Active	58358328.6 (28.9)	17493954.7 (33.7)	14669261.3 (29.7)	13802646.8 (27.3)	12392465.7 (24.7)	
Insufficiently	46854957.4 (23.2)	11901223.9 (22.9)	11127554.4 (22.5)	12120485.6 (24.0)	11705693.5 (23.3)	
Inactive	83188212.5 (41.2)	18905683.3 (36.4)	20173830.4 (40.8)	21334164.5 (42.2)	22774534.3 (45.4)	
Missing data ^c^	13754933.0 (6.8)	3670700.8 (7.1)	3469531.9 (7.0)	3303809.2 (6.5)	3310891.1 (6.6)	
CVD (%)						<0.001
No	184957521.7 (91.5)	49261629.1 (94.8)	45836575.0 (92.7)	45831107.8 (90.6)	44028209.8 (87.7)	
Yes	17184657.3 (8.5)	2709251.1 (5.2)	3600701.2 (7.3)	4725269.7 (9.3)	6149435.2 (12.3)	
Missing data ^c^	14252.6 (0.0)	682.6 (0.0)	2901.9 (0.0)	4728.6 (0.0)	5939.5 (0.0)	

a Values are mean (standard deviation) for continuous variables and percentages for categorical variables.

b Numbers may not sum to the total number of participants due to missing data.

c The total did not sum to 100% because small proportions of participants chose “prefer not to answer” or “do not know”.

### Relationships of UHR level with mortality

**Table 2** presents the results of Cox regression analysis, revealing a positive association between UHR levels and all-cause and CVD mortality, adjusting for covariates. No significant association was found between UHR and malignant neoplasms mortality. The hazard ratios (HRs) and 95% confidence intervals (CIs) in the highest quintile were 1.16 (95% CI: 1.05, 1.29) for all-cause mortality and 1.2 (95% CI: 1.00, 1.45) for CVD mortality, indicating increasing risk with higher UHR quartiles. Interestingly, not all the quadratic term for UHR was not statistically significant, suggesting a non-linear association between UHR and mortality.

**Table 2:** Associations of serum UHR with mortality in US adults.

All-cause mortality	Mode 1			Mode 2			Mode 3		
	OR	95%CI	*P*	OR	95%CI	*P*	OR	95%CI	*P*
UHR	1.03	1.03,1.04	<0.001	1.03	1.02,1.04	<0.001	1.02	1.01,1.02	<0.001
Q1	Reference								
Q2	1.16	1.07,1.26	0.0002	0.99	0.91,1.08	0.895	0.91	0.83,0.99	0.0366
Q3	1.24	1.14,1.34	<0.001	1.03	0.95,1.13	0.478	0.93	0.84,1.02	0.1182
Q4	1.57	1.45,1.71	<0.001	1.39	1.26,1.54	<0.001	1.16	1.05,1.29	0.0052
*P* for trend			<0.001			<0.001			0.0007
CVD mortality									
UHR	1.05	1.03,1.06	<0.001	1.05	1.04,1.06	<0.001	1.03	1.02,1.04	<0.001
Q1	Reference								
Q2	1.17	1,1.37	0.0525	0.99	0.84,1.16	0.8633	0.82	0.69,0.98	0.026
Q3	1.44	1.23,1.68	<0.001	1.2	1.02,1.41	0.0301	0.94	0.79,1.12	0.4795
Q4	1.97	1.68,2.31	<0.001	1.76	1.48,2.08	<0.001	1.2	1,1.45	0.0463
*P* for trend			<0.001			<0.001			0.0038
Malignant neoplasms mortality									
UHR	1.03	1.02,1.04	<0.001	1.01	1,1.03	0.0299	1.01	1,1.02	0.169
Q1	Reference								
Q2	1.16	0.95,1.42	0.1446	0.94	0.77,1.15	0.563	0.9	0.74,1.09	0.27
Q3	1.32	1.11,1.57	0.0018	0.97	0.81,1.17	0.771	0.92	0.76,1.12	0.424
Q4	1.49	1.23,1.8	<0.001	1.1	0.91,1.34	0.319	1.01	0.82,1.24	0.94
*P* for trend			<0.001			0.247			0.746

multivariate weighted Cox proportional hazards regression models with three models to control for confounding factors.

Model 1 was unadjusted;

Model 2 was adjusted for age, gender and race;

Model 3 was adjusted for age, gender, race, education level, family income level, BMI, smoking status, alcohol intake, diabetes and hypertension.

Patients in the highest quintile of diabetes, obesity, and CVD exhibited significantly elevated risks for both all-cause and CVD mortality compared to those in the lowest quintile. Specifically, HRs were 1.32 (95% CI: 1.11, 1.57) for all-cause mortality and 1.38 (95% CI: 1.01, 1.9) for CVD mortality in diabetes patients (**Supplementary Table 4**); 1.32 (95% CI: 1.10, 1.58) and 1.55 (95% CI: 1.06, 2.28) in obesity patients (**Supplementary Table 5**); and 1.29 (95% CI: 1.10, 1.50) and 1.38 (95% CI: 1.06, 1.79) in CVD patients (**Supplementary Table 6**), respectively.

### The dose-response association of UHR level with mortality

**Figure 2:**
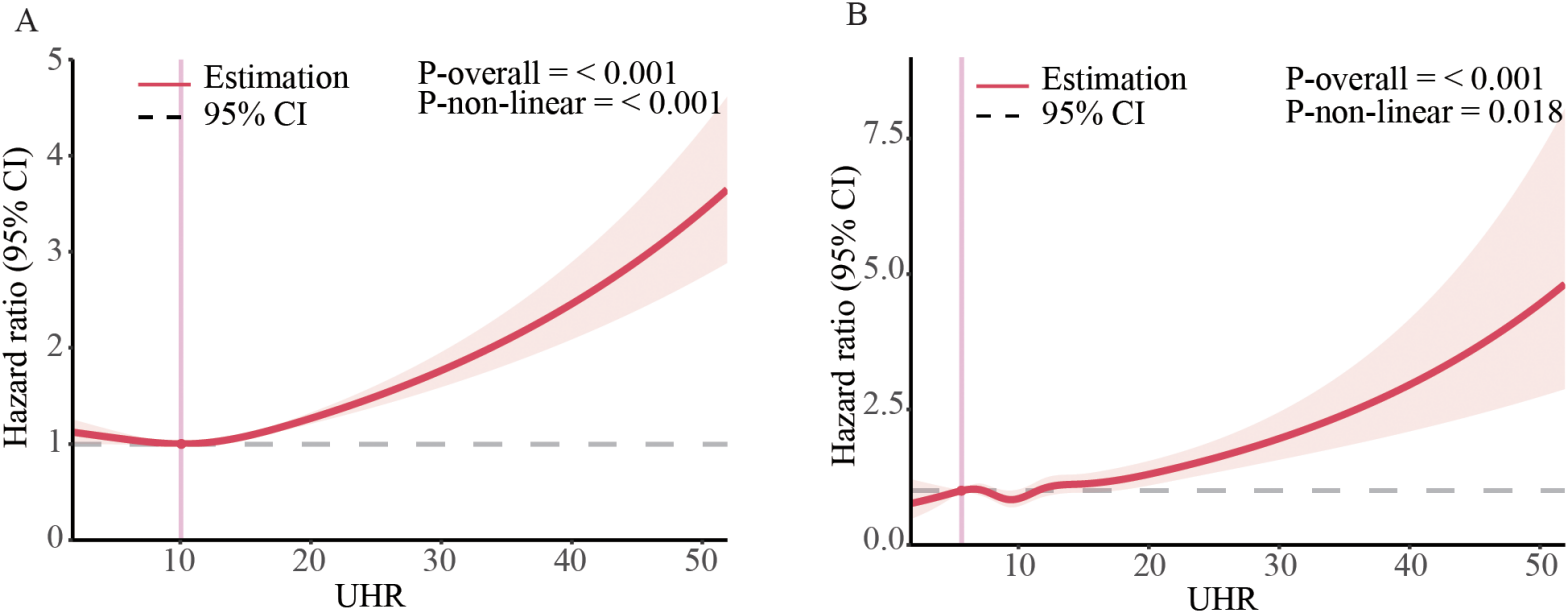
Dose-response associations of UHR with all-cause, and CVD mortality in US adults. Association between UHR and all-cause **(A)** and CVD mortality **(B)** in general population. The associations were examined by multivariable Cox regression models with restricted cubic splines. HRs adjusted for age, gender, race, education level, family income level, BMI, smoking status, alcohol intake, diabetes and hypertension except the corresponding stratification variable. Solid lines represent estimates of HRs and dashed lines represent 95% CIs.

Due to Cox regression analysis indicated a non-linear relationship between UHR and the risk of all-cause and CVD mortality, we employed a restricted cubic splines models to further investigate the correlation. After adjusting for multiple potential confounders, we found a non-linear relationship between UHR and all-cause (*P*-nonlinearity < 0.0001) (**Figure 2A**) and CVD (*P*-nonlinearity = 0.018) (**Figure 2B**) in general population and different subgroup (**Supplementary Figure 1**).

Using Kaplan-Meier analysis, we identified critical thresholds of 12.4 for all-cause mortality and 10.7 for CVD mortality. Analysis of adjusted Cox-regression survival estimates across UHR groups, stratified by these thresholds, demonstrated significant dose-dependent increases in both all-cause (adjusted HR: 1.16, 95% CI: 1.09, 1.24, *P* < 0.0011) and CVD mortality (adjusted HR: 1.2, 95% CI: 1.05, 1.37, *P* = 0.0064). Notably, survival rates were notably lower in the high UHR group, as summarized in **Table 3**.

**Table 3:** Threshold effect analysis of UHR on mortality in US patients.

General population			
All-cause mortality	HR	95% CI	*P* value
UHR	1.02	1.02,1.03	<0.001
Low group: UHR<12.4	Reference		
High group: UHR ≥12.4	1.16	1.09,1.24	<0.001
CVD mortality			
UHR	1.03	1.02,1.04	<0.001
Low group:UHR<10.7	Reference		
High group: UHR ≥10.7	1.2	1.05,1.37	0.0064

Cox proportional hazards models were used to estimate HR and 95% CI. Adjusted for age, gender, race, education level, family income level, BMI, smoking status, alcohol intake, diabetes and hypertension.

In the subgroup analysis, adjusted Cox-regression survival analysis demonstrated significant and dose-dependent increases in both all-cause and CVD mortality among UHR groups stratified by individual thresholds. Specifically, for the diabetes subgroup, the HRs were 1.32 (95% CI: 1.15, 1.51) for all-cause mortality and 1.5 (95% CI: 1.18, 1.89) for CVD mortality compared to the low group (**Supplementary Table 7**). Comparable trends were observed in the obese and CVD subgroups, with adjusted HRs of 1.22 (95% CI: 1.08, 1.37) for all-cause mortality and 1.49 (95% CI: 1.17, 1.9) for CVD mortality in obese patients (**Supplementary Table 8**), and 1.37 (95% CI: 1.16, 1.62) for all-cause mortality and 1.46 (95% CI: 1.21, 1.77) for CVD mortality in CVD patients (**Supplementary Table 9**).

### Stratified analyses

**Figure 3:**
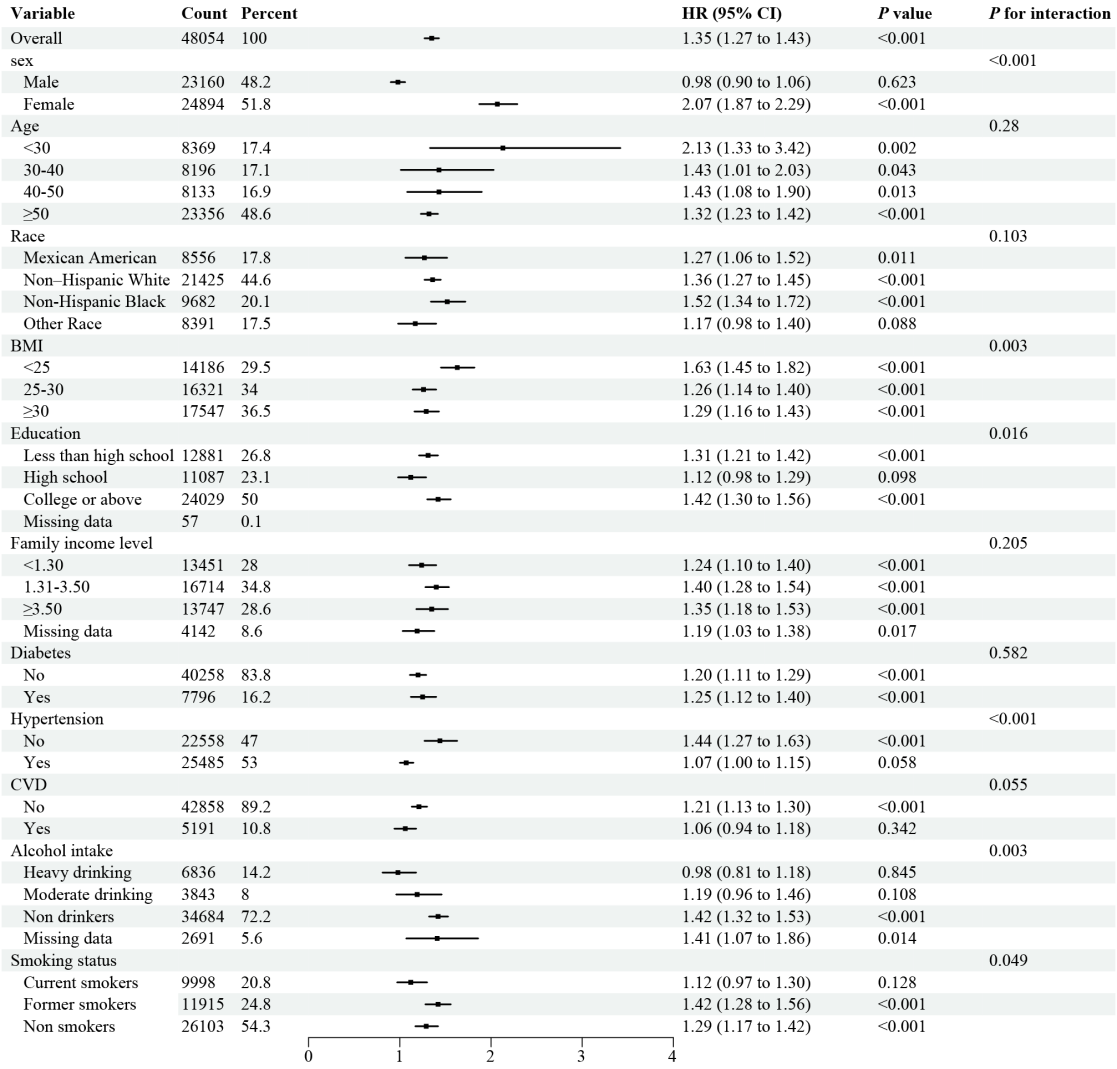
Forest plot of Stratified analyses of UHA and all-cause mortality in general population. HRs adjusted for age, gender, race, education level, family income level, BMI, smoking status, alcohol intake, diabetes and hypertension except the corresponding stratification variable.

**Figure 4:**
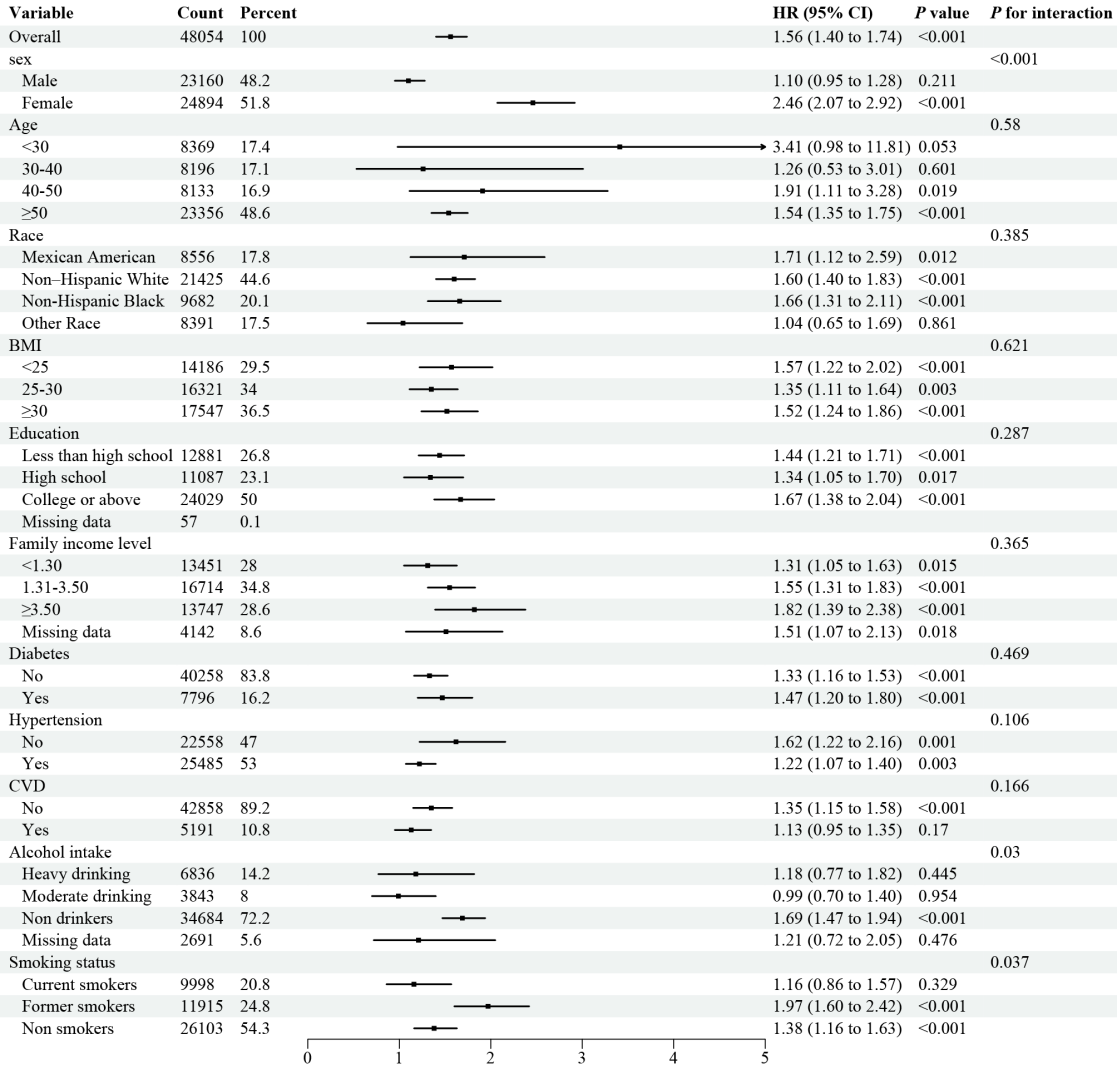
Forest plot of Stratified analyses of UHA and CVD mortality in general population. HRs adjusted for age, gender, race, education level, family income level, BMI, smoking status, alcohol intake, diabetes and hypertension except the corresponding stratification variable.

Stratified analyses demonstrated the disadvantage of higher UHR (≥ 12.4 for all-cause mortality) versus lower UHR (< 12.4) was consistent across subgroups in the general population (**Figure 3**). Significant interactions were observed between UHR and stratified variables, particularly gender, BMI, education, smoking, alcohol use, and hypertension. subgroup analysis revealed a strong association between UHR and all-cause mortality among female patients aged over 50. Similarly, the disadvantage of higher UHR (≥ 10.7 for CVD mortality) compared to lower UHR (< 10.7) was consistent across subgroups (**Figure 4**), with significant interactions based on gender, smoking, and alcohol intake. These findings underscore the importance of considering multiple factors when assessing the impact of UHR on mortality outcomes.

The stratified analyses on diabetes subgroup model revealed significant interactions between sex groups for all-cause mortality, and age categories, education level, and smoking status for CVD mortality (**Supplementary Figures 2**, **3**). The stratified analyses on obesity subgroup model revealed significant interactions between sex groups, education level, and smoking status for all-cause mortality, and sex groups, education level, and diabetes status for CVD mortality (**Supplementary Figures 4**, **5**). The stratified analyses on CVD subgroup model revealed significant interactions between sex groups and education level for all-cause mortality, and education level for CVD mortality (**Supplementary Figures 6**, **7**).

## Discussion

To our knowledge, this is the first study to investigate the relationship between the UHR with all-cause and CVD mortality among US general population, the current study demonstrated that higher UHR levels are associated with increased risks of both types of mortality. Kaplan-Meier analysis identified thresholds of 12.4 and 10.7 for all-cause and CVD mortality, respectively. Our results underscore the predictive power of UHR for cardiovascular and overall mortality.

Studies conducted by Yu et al. have investigated the association between UHR and all-cause/CVD mortality among peritoneal dialysis (PD) patients. Their findings revealed that patients with higher UHR exhibited an elevated risk of both all-cause and cardiovascular mortality, particularly among those aged 65 and older (39). Our study aligns with this evidence, further validating the predictive value of UHR for mortality. Meanwhile, previous studies have shown that UHR is a reliable marker for CVD risk across patient groups. Elevated UHR predicts increased risk of adverse cardiovascular events and CVD mortality, as demonstrated by studies on acute myocardial infarction (22), coronary chronic total occlusion (19), and ischemic heart disease (17). Our study further confirms the correlation between UHR and risk of CVD mortality across diverse patient groups, emphasizing the significance of UHR as a predictor of cardiovascular disease outcomes.

Although the precise biological mechanisms linking UHR index to mortality remain elusive, insulin resistance (IR) is a potential key pathway. IR, characterized by reduced insulin sensitivity and responsiveness, can trigger oxidative stress, exacerbate inflammation, promote foam cell formation, impair endothelial function, and encourage smooth muscle cell proliferation (48). Persistent IR can also increase sympathetic nervous system activity, renal sodium retention, and blood pressure, leading to vascular and renal damage (49). These pathological changes contribute to CVD development, progression, and poor prognosis. Multiple studies suggest a correlation between UHR and IR, with Xu et al. (26) finding a positive association between UHR and HOMA-IR in type 2 diabetes patients, and Dağ et al. (50) observing a link between high UHR levels and obesity/IR in adolescents. This suggests that UHR may influence all-cause and cardiovascular mortality through IR. Meanwhile, our study, in alignment with numerous previous investigations (21, 26–28, 30, 31, 50, 51), further confirms that an increase in UHR corresponds to a gradual elevation in multiple risk factors for CVD and IR, including BMI, FBG, HbA1c, TG, TC and LDL. Additionally, UHR has been associated with a spectrum of metabolic-inflammatory diseases, ranging from diabetes mellitus (31, 52), metabolic syndrome (53), and NAFLD (35, 36, 38, 54). Collectively, these findings suggest that the association between UHR and adverse outcomes is primarily explained by the presence of traditional CVD and IR risk factors.

We further studied the population with diabetes, obesity, and CVD separately, and the results showed that there was still a nonlinear relationship between the UHR index and the all-cause mortality and CVD mortality of the population with diabetes, obesity, and CVD. Among these different populations, the diagnostic predictive value of UHR is higher than that of the general population. Taken together, our findings support the utility of the UHR as a reliable and accurate indicator of all-cause and CVD mortality in the real world.

To fully appreciate the research findings, acknowledging the limitations of this cross-sectional study is paramount. Firstly, causality cannot be definitively established, necessitating further cohort studies to confirm the results. Secondly, cross-sectional studies, although valuable, are susceptible to confounding variables that could potentially bias the results, thereby affecting the interpretation of the findings. Although attempts were made to account for these factors, unknown variables or biases may still exist, leading to inaccurate results. Therefore, a cautious approach is warranted when interpreting the findings, requiring further validation under different conditions. At last, this analysis only examines the prognostic value of the UHR, and it is unclear whether changes in the UHR during follow-up also predict mortality, which requires further investigation.

In conclusion, our study highlights the UHR as a key predictor of all-cause and CVD mortality across different populations. Measuring UHR could aid risk assessment and prognosis. Future research should explore interventions targeting UHR for improved outcomes.

## Data Availability

Publicly available datasets were analyzed in this study. This data can be found here: https://www.cdc.gov/nchs/nhanes/index.htm.
