# Supplementary material for: The serum uric acid-to-high-density lipoprotein cholesterol ratio is a predictor for all-cause and cardiovascular disease mortality: a cross-sectional study
Source: Front Endocrinol (Lausanne). 2024 Sep 13;15:1417485. doi: 10.3389/fendo.2024.1417485 (PMC11427315; doi:10.3389/fendo.2024.1417485)
Supplement: Supplementary file 1 [file DataSheet1.pdf]

Table S1. Baseline characteristics of patients with diabetes.

Patients with diabetes	Overall	The UHR quartiles [a]				*P*
		≤9.08	9.08-12.09	12.09-15.95	> 15.95	
n (cases)	24154251.61	5731094.36	5802397.14	6329530.14	6291229.97	
Gender [Female (%)]	11627563.1(48.1)	4044716.8(70.6)	3212183.6(55.4)	2436358.7(38.5)	1934303.9(30.7)	<0.001
Age (years)	58.95 ± 13.93	58.71 ± 14.49	59.14 ± 13.86	58.89 ± 13.19	59.06 ± 14.19	0.886
Race (%)						<0.001
Mexican American	2251470.0(9.3)	618152.4(10.8)	620459.3(10.7)	565942.4(8.9)	446915.9(7.1)	
Other race	3504592.0(14.5)	923505.6(16.1)	867191.7(14.9)	858948.3(13.6)	854946.5(13.6)	
Non–Hispanic White	14887467.8(61.6)	3186019.4(55.6)	3416759.0(58.9)	4100601.7(64.8)	4184087.8(66.5)	
Non-Hispanic Black	3510721.7(14.5)	1003417.0(17.5)	897987.2(15.5)	804037.8(12.7)	805279.7(12.8)	
Education (%)						0.663
Less than high school	6158082.6(25.5)	1498510.0(26.1)	1557403.9(26.8)	1514439.6(23.9)	1587729.2(25.2)	
High school	6075894.7(25.2)	1454363.5(25.4)	1426368.3(24.6)	1547740.3(24.5)	1647422.5(26.2)	
College or above	11904361.8(49.3)	2774585.6(48.4)	2815544.0(48.5)	3260415.7(51.5)	3053816.5(48.5)	
Missing data [c]	15912.5(0.1)	3635.2(0.1)	3081.1(0.1)	6934.5(0.1)	2261.7(0.0)	
PIR (%)						0.34
< 1.30	5669604.1(23.5)	1361051.7(23.7)	1355204.1(23.4)	1423654.4(22.5)	1529694.0(24.3)	
1.31-3.50	8828967.1(36.6)	2139063.5(37.3)	2069264.9(35.7)	2221172.5(35.1)	2399466.3(38.1)	
≥3.50	7718634.2(32.0)	1717295.1(30.0)	1868732.7(32.2)	2208250.9(34.9)	1924355.5(30.6)	
Missing data [c]	1937046.2(8.0)	513684.1(9.0)	509195.4(8.8)	476452.4(7.5)	437714.2(7.0)	
All-cause mortality (%)	5861994.1(24.3)	1271618.0(22.2)	1324882.7(22.8)	1406393.4(22.2)	1859100.1(29.6)	<0.001
CVD mortality (%)	1665962.6(6.9)	360962.7(6.3)	316876.0(5.5)	410362.3(6.5)	577761.6(9.2)	0.001
Malignant neoplasms mortality (%)	955965.9(4.0)	196384.0(3.4)	224675.1(3.9)	261009.6(4.1)	273897.1(4.4)	0.595
Time(years)	8.05 ± 5.17	8.20 ± 5.31	8.17 ± 5.21	7.95 ± 5.04	7.9 ± 5.12	0.55
UHR (%)	13.2 ± 5.63	7.03 ± 1.49	10.59 ± 0.87	13.89 ± 1.14	20.56 ± 4.72	<0.001
ALB (g/L)	41.42 ± 3.5	41.22 ± 3.64	41.28 ± 3.46	41.67 ± 3.39	41.46 ± 3.49	0.004
ALT (U/L)	27.78 ± 27.01	23.34 ± 14.21	27.30 ± 22.97	29.13 ± 26.34	30.92 ± 37.45	<0.001
AST (U/L)	26.38 ± 19.44	24.05 ± 12.41	26.23 ± 20.04	26.41 ± 15.44	28.63 ± 26.28	<0.001
Tbil (μmol/L)	11 ± 5.18	10.38 ± 4.92	10.82 ± 5.38	11.31 ± 5.2	11.42 ± 5.15	<0.001
GGT (IU/L)	37.73 ± 59.9	32.43 ± 42.92	40.11 ± 92.64	36.34 ± 39.89	41.77 ± 50.37	<0.001
LDH(IU/L)	138.99 ± 34.83	139.81 ± 31.71	138.88 ± 35.3	137.41 ± 34.91	139.94 ± 36.94	0.361
BUN (mmol/L)	5.75 ± 2.74	5.16 ± 2.18	5.48 ± 2.34	5.74 ± 2.55	6.55 ± 3.44	<0.001
Scr (μmol/L)	85.39 ± 52.26	75.39 ± 61.42	82.57 ± 55.32	85.86 ± 37.99	96.62 ± 50.56	<0.001
eGFR (mL/min/1.73 m2)	93.86 ± 34.51	105.16 ± 37.11	96.14 ± 33.84	91.42 ± 31.36	83.91 ± 32.35	<0.001

TC (mmol/L)	4.9 ± 1.26	5.03 ± 1.16	4.96 ± 1.31	4.82 ± 1.23	4.8 ± 1.34	<0.001
TG (mmol/L) [b]	1.98 ± 2.05	1.39 ± 1.82	1.75 ± 1.44	2.07 ± 1.6	2.66 ± 2.82	<0.001
LDL-C (mmol/L) [b]	2.74 ± 0.97	2.73 ± 0.94	2.85 ± 0.95	2.75 ± 1.02	2.63 ± 0.97	0.009
BMI (kg/m2)	32.92 ± 7.58	30.39 ± 7.04	32.35 ± 7.24	33.50 ± 7.39	35.15 ± 7.79	<0.001
HbA1c (%)	7.23 ± 1.69	7.36 ± 1.98	7.31 ± 1.76	7.10 ± 1.46	7.18 ± 1.54	0.001
FPG (mmol/L) [b]	8.67 ± 3.39	8.73 ± 3.65	8.92 ± 3.68	8.53 ± 3.09	8.53 ± 3.13	0.146
Hypertension (%)						<0.001
No	5354566.4(22.2)	1613105.3(28.1)	1389327.0(23.9)	1287162.0(20.3)	1064972.1(16.9)	
Yes	18794967.3(77.8)	4113271.2(71.8)	4413070.1(76.1)	5042368.1(79.7)	5226257.9(83.1)	
Missing data [c]	4717.9(0.0)	4717.9(0.1)	0.0(0.0)	0.0(0.0)	0.0(0.0)	
Smoking status (%)						<0.001
Current smokers	4038392.6(16.7)	906442.4(15.8)	971903.0(16.8)	974115.7(15.4)	1185931.5(18.9)	
Former smokers	8334088.5(34.5)	1673099.8(29.2)	1811532.1(31.2)	2310710.7(36.5)	2538745.9(40.4)	
Nonsmokers	11765207.5(48.7)	3149624.5(55.0)	3018261.2(52.0)	3039715.1(48.0)	2557606.7(40.7)	
Missing data [c]	16562.9(0.1)	1927.7(0.0)	700.8(0.0)	4988.7(0.1)	8945.7(0.1)	
Alcohol consumption (%)						0.002
Heavy drinking	2083734.5(8.6)	653210.5(11.4)	414857.2(7.1)	586006.3(9.3)	429660.5(6.8)	
Moderate drinking	1533149.4(6.3)	279520.8(4.9)	350417.0(6.0)	456544.6(7.2)	446667.0(7.1)	
Nondrinkers	19205076.6(79.5)	4406539.2(76.9)	4690280.6(80.8)	4985316.1(78.8)	5122940.7(81.4)	
Missing data [c]	1332291.0(5.5)	391823.8(6.8)	346842.3(6.0)	301663.1(4.8)	291961.8(4.6)	
Physical activity (%)						0.072
Active	4300382.1(17.8)	1111397.1(19.4)	1002997.1(17.3)	1185399.0(18.7)	1000588.9(15.9)	
Insufficiently	4537011.2(18.8)	1130943.1(19.7)	1169027.3(20.1)	1148341.6(18.1)	1088699.2(17.3)	
Inactive	13662878.9(56.6)	2999047.5(52.3)	3280201.6(56.5)	3594607.4(56.8)	3789022.5(60.2)	
Missing data [c]	1653979.4(6.8)	489706.7(8.5)	350171.1(6.0)	401182.2(6.3)	412919.4(6.6)	
CVD (%)						<0.001
No	18339866.8(75.9)	4648219.0(81.1)	4489587.2(77.4)	4838594.6(76.4)	4363465.9(69.4)	
Yes	5811545.6(24.1)	1082192.7(18.9)	1312809.9(22.6)	1488778.9(23.5)	1927764.1(30.6)	
Missing data [c]	2839.2(0.0)	682.6(0.0)	0.0(0.0)	2156.6(0.0)	0.0(0.0)	

[a] Values are mean (standard deviation) for continuous variables and percentages for categorical variables.

[b] Numbers may not sum to the total number of participants due to missing data.

[c] The total number did not sum to 100% because small proportions of participants chose "prefer not to answer" or "do not know".

Table S2. Baseline characteristics of patients with obesity.

Patients with obesity	Overall	The UHR quartiles [a]				P
		≤9.07	9.07-12.22	12.22-16.18	> 16.18	
n (cases)	71664710.2	16259995.53	18040861.57	18451008.36	18912844.73	
Gender [Female (%)]	38631037.6(53.9)	14397801.1(88.5)	12066061.7(66.9)	8081258.4(43.8)	4085916.4(21.6)	<0.001
age (years)	47.98 ± 15.82	48.38 ± 15.81	48.9 ± 15.87	48.62 ± 15.44	46.15 ± 16.01	<0.001
Race (%)						<0.001
Mexican American	6888214.6(9.6)	1655582.9(10.2)	1815732.4(10.1)	1756713.9(9.5)	1660185.3(8.8)	
Other race	7284702.7(10.2)	1643810.9(10.1)	1662267.7(9.2)	1953327.7(10.6)	2025296.4(10.7)	
Non–Hispanic White	47585640.2(66.4)	9801860.8(60.3)	11921828.3(66.1)	12493825.6(67.7)	13368125.4(70.7)	
Non-Hispanic Black	9906152.7(13.8)	3158740.9(19.4)	2641033.2(14.6)	2247141.0(12.2)	1859237.7(9.8)	
Education (%)						<0.001
Less than high school	12772936.1(17.8)	2799689.6(17.2)	3048102.1(16.9)	3352322.5(18.2)	3572821.8(18.9)	
High school	18623509.6(26.0)	3784869.2(23.3)	4741214.8(26.3)	4856727.5(26.3)	5240698.1(27.7)	
College or above	40224926.0(56.1)	9667235.7(59.5)	10248644.9(56.8)	10237402.4(55.5)	10071643.0(53.3)	
Missing data [c]	43338.6(0.1)	8201.0(0.1)	2899.7(0.0)	4556.0(0.0)	27681.8(0.1)	
PIR (%)						0.384
< 1.30	14925538.9(20.8)	3542621.0(21.8)	3649693.7(20.2)	3718288.3(20.2)	4014936.0(21.2)	
1.31-3.50	25365849.2(35.4)	5448603.8(33.5)	6589213.9(36.5)	6686427.4(36.2)	6641604.1(35.1)	
≥3.50	26497489.3(37.0)	6070508.5(37.3)	6576760.1(36.5)	6746811.3(36.6)	7103409.3(37.6)	
Missing data [c]	4875832.8(6.8)	1198262.1(7.4)	1225194.0(6.8)	1299481.3(7.0)	1152895.4(6.1)	
All-cause mortality (%)	7715125.5(10.8)	1342995.0(8.3)	1826136.3(10.1)	2026262.8(11.0)	2519731.5(13.3)	<0.001
CVD mortality (%)	2150652.2(3.0)	356120.9(2.2)	439068.4(2.4)	581827.0(3.2)	773635.8(4.1)	<0.001
Malignant neoplasms mortality (%)	1785357.4(2.5)	298168.3(1.8)	494962.6(2.7)	472152.1(2.6)	520074.5(2.7)	0.061
Time(years)	9.37 ± 5.58	9.26 ± 5.66	9.13 ± 5.59	9.41 ± 5.47	9.65 ± 5.61	0.005
UHR (%)	13.36 ± 5.53	7.19 ± 1.38	10.63 ± 0.9	14.05 ± 1.16	20.61 ± 4.41	<0.001
ALB (g/L)	41.66 ± 3.38	40.53 ± 3.59	41.46 ± 3.19	42 ± 3.24	42.48 ± 3.24	<0.001
ALT (U/L)	28.52 ± 22.35	22.03 ± 15	25.42 ± 18.47	29.91 ± 21.17	35.7 ± 28.96	<0.001
AST (U/L)	25.49 ± 15.71	22.62 ± 11.91	24.29 ± 17.75	25.88 ± 12.89	28.72 ± 18.21	<0.001
Tbil (μmol/L)	10.65 ± 5.02	9.38 ± 4.51	10.24 ± 4.9	11.05 ± 5.11	11.75 ± 5.17	<0.001
GGT (IU/L)	32.46 ± 40.63	26.81 ± 37.47	30.79 ± 51.12	33.21 ± 34.38	38.18 ± 36.6	<0.001
LDH(IU/L)	138.31 ± 30.91	138.43 ± 30.78	138.12 ± 32.03	138.01 ± 29.64	138.67 ± 31.14	0.87
BUN (mmol/L)	4.91 ± 2.05	4.48 ± 1.75	4.77 ± 1.82	4.96 ± 2.01	5.35 ± 2.41	<0.001
Scr (μmol/L)	77.98 ± 33.99	67.34 ± 29.59	73.85 ± 25.75	80.76 ± 38.33	88.38 ± 36.47	<0.001
eGFR (mL/min/1.73 m2)	100.82 ± 31.39	110.73 ± 36.62	102.6 ± 30.73	97.54 ± 27.72	93.79 ± 27.99	<0.001

TC (mmol/L)	5.1 ± 1.08	5.2 ± 1.02	5.11 ± 1.04	5.07 ± 1.09	5.06 ± 1.13	<0.001
TG (mmol/L) [b]	1.75 ± 1.52	1.23 ± 1.22	1.5 ± 1.04	1.78 ± 1.12	2.42 ± 2.13	<0.001
LDL-C(mmol/L) [b]	3.03 ± 0.91	2.99 ± 0.87	3.04 ± 0.89	3.08 ± 0.94	3.01 ± 0.92	0.104
BMI (kg/m2)	35.84 ± 5.69	35.12 ± 5.11	35.71 ± 5.52	36.08 ± 5.95	36.33 ± 5.97	<0.001
HbA1c (%)	5.82 ± 1.08	5.7 ± 1.07	5.84 ± 1.13	5.86 ± 1.03	5.86 ± 1.08	<0.001
FPG (mmol/L) [b]	6.27 ± 2.05	5.92 ± 1.84	6.31 ± 2.18	6.36 ± 1.98	6.43 ± 2.11	<0.001
Diabetes (%)						<0.001
No	56931101.2(79.4)	13687482.0(84.2)	14404184.7(79.8)	14273484.0(77.4)	14565950.5(77.0)	
Yes	14733609.0(20.6)	2572513.5(15.8)	3636676.9(20.2)	4177524.3(22.6)	4346894.3(23.0)	
Hypertension (%)						<0.001
No	27116250.6(37.8)	7578731.6(46.6)	6729258.8(37.3)	6578135.9(35.7)	6230124.4(32.9)	
Yes	44546686.9(62.2)	8679491.3(53.4)	11311602.8(62.7)	11872872.5(64.3)	12682720.3(67.1)	
Missing data [c]	1772.7(0.0)	1772.7(0.0)	0.0(0.0)	0.0(0.0)	0.0(0.0)	
Smoking status (%)						<0.001
Current smokers	13221798.1(18.4)	2426581.8(14.9)	3194506.4(17.7)	3334361.4(18.1)	4266348.5(22.6)	
Former smokers	19551475.3(27.3)	3836980.4(23.6)	4615293.9(25.6)	5551830.3(30.1)	5547370.7(29.3)	
Nonsmokers	38854610.9(54.2)	9980978.3(61.4)	10230093.2(56.7)	9559828.0(51.8)	9083711.4(48.0)	
Missing data [c]	36825.8(0.1)	15455.0(0.1)	968.1(0.0)	4988.7(0.0)	15414.1(0.1)	
Alcohol consumption (%)						<0.001
Heavy drinking	9613556.4(13.4)	2299819.8(14.1)	2446327.7(13.6)	2437696.6(13.2)	2429712.3(12.8)	
Moderate drinking	4707517.4(6.6)	673365.8(4.1)	1079849.3(6.0)	1331587.9(7.2)	1622714.3(8.6)	
Nondrinkers	54323600.1(75.8)	12512644.2(77.0)	13685297.1(75.9)	13967655.9(75.7)	14158003.0(74.9)	
Missing data [c]	3020036.3(4.2)	774165.7(4.8)	829387.5(4.6)	714068.0(3.9)	702415.1(3.7)	
Physical activity (%)						0.717
Active	16482925.4(23.0)	3929040.0(24.2)	4149287.2(23.0)	4128530.1(22.4)	4276068.1(22.6)	
Insufficiently	16459480.4(23.0)	3490267.0(21.5)	4149970.0(23.0)	4421868.8(24.0)	4397374.5(23.3)	
Inactive	34726138.9(48.5)	7877817.7(48.4)	8806055.3(48.8)	8843815.6(47.9)	9198450.3(48.6)	
Missing data [c]	3996165.5(5.6)	962870.8(5.9)	935549.0(5.2)	1056793.8(5.7)	1040951.9(5.5)	
CVD (%)						<0.001
No	63904193.4(89.2)	14906243.9(91.7)	16392584.9(90.9)	16255112.6(88.1)	16350252.0(86.5)	
Yes	7754577.3(10.8)	1353751.7(8.3)	1648276.7(9.1)	2193739.1(11.9)	2558809.8(13.5)	
Missing data [c]	5939.5(0.0)	0.0(0.0)	0.0(0.0)	2156.6(0.0)	3782.9(0.0)	

[a] Values are mean (standard deviation) for continuous variables and percentages for categorical variables.

[b] Numbers may not sum to the total number of participants due to missing data.

[c] The total number did not sum to 100% because small proportions of participants chose "prefer not to answer" or "do not know".

Table S3. Baseline characteristics of patients with CVD.

Patients with CVD	Overall	The UHR quartiles [a]				P
		≤9.04	9.04-12.38	12.38-16.25	> 16.25	
n (cases)	17184657.3	4481492.86	4227885.57	4305914.28	4169364.59	
Gender [Female (%)]	7934553.0(46.2)	3207359.8(71.6)	2152790.1(50.9)	1531072.4(35.6)	1043330.6(25.0)	<0.001
Age (years)	64.61 ± 13.56	64.8 ± 14.32	65.24 ± 13.45	64.19 ± 12.89	64.21 ± 13.51	0.326
Race (%)						0.496
Mexican American	714733.6(4.2)	179329.3(4.0)	176745.1(4.2)	207486.1(4.8)	151173.2(3.6)	
Other race	1586371.6(9.2)	423666.2(9.5)	376403.5(8.9)	395206.8(9.2)	391095.0(9.4)	
Non–Hispanic White	13019913.6(75.8)	3340819.2(74.5)	3186832.2(75.4)	3261024.3(75.7)	3231237.9(77.5)	
Non-Hispanic Black	1863638.5(10.8)	537678.2(12.0)	487904.8(11.5)	442197.0(10.3)	395858.5(9.5)	
Education (%)						0.369
Less than high school	4543668.1(26.4)	1126789.1(25.1)	1106747.6(26.2)	1183508.7(27.5)	1126622.7(27.0)	
High school	4670494.4(27.2)	1194556.7(26.7)	1142580.8(27.0)	1080298.0(25.1)	1253059.0(30.1)	
College or above	7954864.3(46.3)	2154302.6(48.1)	1973509.3(46.7)	2038697.5(47.3)	1788354.9(42.9)	
Missing data [c]	15630.5(0.1)	5844.4(0.1)	5047.9(0.1)	3410.1(0.1)	1328.0(0.0)	
PIR (%)						0.252
< 1.30	4463650.1(26.0)	1231394.8(27.5)	1045450.5(24.7)	1093887.7(25.4)	1092917.1(26.2)	
1.31-3.50	6517623.7(37.9)	1728155.3(38.6)	1685802.4(39.9)	1482519.2(34.4)	1621146.8(38.9)	
≥3.50	4905459.7(28.5)	1222677.1(27.3)	1156584.2(27.4)	1385626.8(32.2)	1140571.6(27.4)	
Missing data [c]	1297923.8(7.6)	299265.7(6.7)	340048.5(8.0)	343880.5(8.0)	314729.1(7.5)	
All-cause mortality (%)	6547975.5(38.1)	1588946.9(35.5)	1625069.0(38.4)	1516419.2(35.2)	1817540.3(43.6)	0.003
CVD mortality (%)	2214143.5(12.9)	528380.7(11.8)	523768.0(12.4)	494992.3(11.5)	667002.5(16.0)	0.013
Malignant neoplasms mortality (%)	1132572.5(6.6)	259821.8(5.8)	263813.1(6.2)	289763.2(6.7)	319174.5(7.7)	0.394
Time(years)	7.9 ± 5.16	7.76 ± 5.05	8.03 ± 5.03	8.19 ± 5.37	7.64 ± 5.18	0.217
UHR (%)	13.11 ± 5.83	6.91 ± 1.48	10.67 ± 0.98	14.13 ± 1.17	21.2 ± 4.65	<0.001
ALB (g/L)	41.52 ± 3.42	41.51 ± 3.44	41.55 ± 3.31	41.67 ± 3.45	41.34 ± 3.46	0.309
ALT (U/L)	24.13 ± 26.28	22.3 ± 16.35	21.99 ± 11.73	25.8 ± 26.21	26.52 ± 41.18	<0.001
AST (U/L)	25.46 ± 17.58	25.4 ± 13.93	24.15 ± 9.68	25.88 ± 15.16	26.44 ± 27.04	0.001
Tbil (μmol/L)	11.53 ± 5.21	10.86 ± 4.76	11.42 ± 5.1	11.61 ± 5.34	12.28 ± 5.54	<0.001
GGT (IU/L)	34.03 ± 44.8	32.05 ± 46.24	32.14 ± 43.98	35.72 ± 45.77	36.33 ± 42.88	0.025
LDH(IU/L)	144.02 ± 36.85	144.97 ± 36.66	143.01 ± 37.04	143.76 ± 36.82	144.27 ± 36.92	0.721
BUN (mmol/L)	6.15 ± 3.05	5.31 ± 2.35	6.01 ± 2.74	6.13 ± 2.81	7.23 ± 3.84	<0.001
Scr (μmol/L)	93.99 ± 59.79	79.72 ± 52.16	94.48 ± 73.49	96.06 ± 61.49	106.68 ± 45.28	<0.001
eGFR (mL/min/1.73 m2)	82.7 ± 29.7	92.89 ± 29.92	82.64 ± 28.59	81.81 ± 29.16	72.69 ± 27.51	<0.001

TC (mmol/L)	4.82 ± 1.19	5.05 ± 1.13	4.81 ± 1.14	4.81 ± 1.17	4.58 ± 1.29	<0.001
TG (mmol/L) [b]	1.69 ± 1.27	1.16 ± 0.71	1.48 ± 0.74	1.87 ± 1.02	2.31 ± 1.93	<0.001
LDL-C (mmol/L) [b]	2.69 ± 0.99	2.73 ± 0.96	2.7 ± 0.96	2.8 ± 1.02	2.52 ± 1	0.002
BMI (kg/m2)	30.27 ± 7.01	27.68 ± 6.57	29.59 ± 6.33	31.13 ± 6.78	32.86 ± 7.31	<0.001
HbA1c (%)	6.07 ± 1.2	5.85 ± 1.14	6.11 ± 1.27	6.06 ± 1.06	6.26 ± 1.27	<0.001
FPG (mmol/L) [b]	6.65 ± 2.42	6.12 ± 2	6.86 ± 2.81	6.63 ± 2.05	7.03 ± 2.6	<0.001
Diabetes (%)						
No	11373111.7(66.2)	3424016.9(76.4)	2779881.6(65.8)	2775755.6(64.5)	2393457.5(57.4)	<0.001
Yes	5811545.6(33.8)	1057475.9(23.6)	1448004.0(34.2)	1530158.7(35.5)	1775907.0(42.6)	
Hypertension (%)						0.311
No	3403112.0(19.8)	964401.2(21.5)	832411.4(19.7)	903470.1(21.0)	702829.3(16.9)	
Yes	13779667.3(80.2)	3515213.6(78.4)	3395474.2(80.3)	3402444.2(79.0)	3466535.3(83.1)	
Missing data [c]	1878.0(0.0)	1878.0(0.0)	0.0(0.0)	0.0(0.0)	0.0(0.0)	
Smoking status (%)						<0.001
Current smokers	3730064.7(21.7)	959315.0(21.4)	1003062.9(23.7)	880730.2(20.5)	886956.7(21.3)	
Former smokers	6843693.5(39.8)	1474634.3(32.9)	1545396.5(36.6)	1834980.9(42.6)	1988681.9(47.7)	
Nonsmokers	6609571.0(38.5)	2047543.6(45.7)	1679426.2(39.7)	1590203.2(36.9)	1292398.0(31.0)	
Missing data [c]	1328.0(0.0)	0.0(0.0)	0.0(0.0)	0.0(0.0)	1328.0(0.0)	
Alcohol consumption (%)						0.02
Heavy drinking	1710390.5(10.0)	558589.3(12.5)	447438.0(10.6)	418459.9(9.7)	285903.3(6.9)	
Moderate drinking	1310571.3(7.6)	277852.9(6.2)	313301.3(7.4)	394013.2(9.2)	325403.8(7.8)	
Nondrinkers	13345711.5(77.7)	3379924.9(75.4)	3254902.0(77.0)	3321823.9(77.1)	3389060.7(81.3)	
Missing data [c]	817984.1(4.8)	265125.7(5.9)	212244.3(5.0)	171617.3(4.0)	168996.8(4.1)	
Physical activity (%)						0.01
Active	3168639.1(18.4)	883507.3(19.7)	803164.3(19.0)	877371.7(20.4)	604595.8(14.5)	
Insufficiently	2958774.4(17.2)	755977.7(16.9)	685177.5(16.2)	828118.4(19.2)	689500.8(16.5)	
Inactive	9330659.5(54.3)	2316900.8(51.7)	2409878.2(57.0)	2171370.3(50.4)	2432510.2(58.3)	
Missing data [c]	1726584.2(10.0)	525107.1(11.7)	329665.6(7.8)	429053.8(10.0)	442757.7(10.6)	

[a] Values are mean (standard deviation) for continuous variables and percentages for categorical variables.

[b] Numbers may not sum to the total number of participants due to missing data.

[c] The total number did not sum to 100% because small proportions of participants chose "prefer not to answer" or "do not know".

Table S4. Associations of serum UHR with mortality in patient with diabetes.

Patient with diabetes	Mode 1			Mode 2			Mode 3		
**All-cause mortality**	**OR**	**95%CI**	***P***	**OR**	**95%CI**	***P***	**OR**	**95%CI**	***P***
**UHR**	1.02	1.01,1.03	0.0003	1.03	1.01,1.04	<0.001	1.02	1.01,1.03	0.0001
**Q1**					Reference				
**Q2**	1.04	0.89,1.21	0.638	1.03	0.89,1.18	0.7072	1.02	0.89,1.18	0.7483
**Q3**	1.05	0.9,1.23	0.506	1.05	0.91,1.22	0.5188	1.03	0.88,1.2	0.6998
**Q4**	1.4	1.19,1.65	0	1.39	1.17,1.64	0.0001	1.32	1.11,1.57	0.0017
***P* for trend**			0.0001			0.0001			0.0019
**CVD mortality**									
**UHR**	1.03	1.01,1.04	0.0004	1.03	1.01,1.05	0.0005	1.03	1.01,1.05	0.0033
**Q1**					Reference				
**Q2**	0.87	0.65,1.18	0.3831	0.86	0.65,1.14	0.3017	0.85	0.63,1.15	0.2941
**Q3**	1.08	0.83,1.41	0.5495	1.07	0.82,1.39	0.6233	1.03	0.78,1.37	0.8279
**Q4**	1.55	1.15,2.08	0.0041	1.49	1.09,2.05	0.0123	1.38	1.01,1.9	0.0459
***P* for trend**			0.0015			0.0045			0.0169

Note: multivariate weighted Cox proportional hazards regression models with three models to control for confounding factors.

Model 1 was unadjusted;

Model 2 was adjusted for age, gender and race;

Model 3 was adjusted for age, gender, race, education level, family income level, BMI, smoking status, alcohol intake, diabetes and hypertension.

Table S5. Associations of serum UHR with mortality in patient with obesity.

Patient with obesity	Mode 1			Mode 2			Mode 3		
**All-cause mortality**	**OR**	**95%CI**	***P***	**OR**	**95%CI**	***P***	**OR**	**95%CI**	***P***
**UHR**	1.03	1.02,1.04	<0.001	1.04	1.03,1.05	<0.001	1.03	1.01,1.04	<0.001
**Q1**					Reference				
**Q2**	1.25	1.06,1.49	0.01	1.09	0.94,1.27	0.257	1.02	0.87,1.19	0.7959
**Q3**	1.31	1.11,1.55	0.0012	1.13	0.95,1.35	0.153	1.04	0.87,1.24	0.662
**Q4**	1.54	1.32,1.79	<0.001	1.57	1.32,1.86	<0.001	1.32	1.1,1.58	0.0025
***P* for trend**			<0.001			<0.001			0.0018
**CVD mortality**									
**UHR**	1.04	1.03,1.06	<0.001	1.06	1.04,1.08	<0.001	1.04	1.02,1.06	<0.001
**Q1**					Reference				
**Q2**	1.14	0.81,1.59	0.4604	0.99	0.71,1.38	0.9518	0.94	0.66,1.32	0.7025
**Q3**	1.42	1.03,1.97	0.0329	1.24	0.86,1.8	0.2517	1.14	0.78,1.67	0.5005
**Q4**	1.78	1.3,2.43	0.0003	1.87	1.29,2.73	0.0011	1.55	1.06,2.28	0.0249
***P* for trend**			<0.001			0.0002			0.006

Note: multivariate weighted Cox proportional hazards regression models with three models to control for confounding factors.

Model 1 was unadjusted;

Model 2 was adjusted for age, gender and race;

Model 3 was adjusted for age, gender, race, education level, family income level, BMI, smoking status, alcohol intake, diabetes and hypertension.

Table S6. Associations of serum UHR with mortality in patient with CVD.

Patient with CVD	Mode 1			Mode 2			Mode 3		
**All-cause mortality**	**OR**	**95%CI**	***P***	**OR**	**95%CI**	***P***	**OR**	**95%CI**	***P***
**UHR**	1.02	1.01,1.03	0.0002	1.02	1.02,1.03	<0.001	1.02	1.01,1.03	<0.001
**Q1**					Reference				
**Q2**	1.04	0.89,1.21	0.6239	1.04	0.91,1.2	0.5426	1.01	0.88,1.16	0.8751
**Q3**	0.92	0.78,1.08	0.323	1.02	0.88,1.18	0.8241	1.02	0.88,1.18	0.8348
**Q4**	1.24	1.06,1.46	0.0068	1.37	1.16,1.61	0.0001	1.29	1.1,1.5	0.0014
***P* for trend**			0.0368			0.0004			0.0021
**CVD mortality**									
**UHR**	1.03	1.01,1.04	<0.001	1.03	1.02,1.05	<0.001	1.03	1.02,1.05	<0.001
**Q1**					Reference				
**Q2**	1.01	0.77,1.33	0.9308	0.99	0.76,1.3	0.9681	0.97	0.74,1.27	0.8168
**Q3**	0.91	0.69,1.19	0.483	0.97	0.73,1.28	0.8221	0.97	0.73,1.28	0.8113
**Q4**	1.37	1.08,1.74	0.0087	1.45	1.13,1.87	0.0041	1.38	1.06,1.79	0.0156
***P* for trend**			0.0245			0.0057			0.0189

Note: multivariate weighted Cox proportional hazards regression models with three models to control for confounding factors.

Model 1 was unadjusted;

Model 2 was adjusted for age, gender and race;

Model 3 was adjusted for age, gender, race, education level, family income level, BMI, smoking status, alcohol intake, diabetes and hypertension.

Table S7. Threshold effect analysis of UHR on mortality in patient with diabetes.

Diabetes patients			
**All-cause mortality**	**HR**	**95% CI**	***P***
UHR	1.02	1.01,1.03	<0.001
Low group: UHR<14.2	Reference		
High group: UHR ≥14.2	1.32	1.15,1.51	0.0001
**CVD mortality**			
UHR	1.03	1.02,1.05	<0.001
Low group: UHR<15.8	Reference		
High group: UHR ≥15.8	1.5	1.18,1.89	0.0009

Note: Cox proportional hazards models were used to estimate HR and 95% CI. Adjusted for age, gender, race, education level, family income level, BMI, smoking status, alcohol intake, diabetes and hypertension.

Table S8. Threshold effect analysis of UHR on mortality in patient with obesity.

Obesity patients			
**All-cause mortality**	**HR**	**95% CI**	***P***
UHR	1.03	1.02,1.04	<0.001
Low group: UHR<13.2	Reference		
High group: UHR ≥13.2	1.22	1.08,1.37	0.0009
**CVD mortality**			
UHR	1.04	1.02,1.06	<0.001
Low group: UHR<13.2	Reference		
High group: UHR ≥13.2	1.49	1.17,1.9	0.0014

Note: Cox proportional hazards models were used to estimate HR and 95% CI. Adjusted for age, gender, race, education level, family income level, BMI, smoking status, alcohol intake, diabetes and hypertension.

Table S9. Threshold effect analysis of UHR on mortality in patient with CVD.

Patient with CVD			
**All-cause mortality**	**HR**	**95% CI**	**P-value**
UHR	1.02	1.01,1.03	<0.001
Low group: UHR<20.7	Reference		
High group: UHR ≥20.7	1.37	1.16,1.62	0.0002
**CVD mortality**			
UHR	1.03	1.02,1.05	0.0001
Low group: UHR<17	Reference		
High group: UHR ≥17	1.46	1.21,1.77	<0.001

Note: Cox proportional hazards models were used to estimate HR and 95% CI. Adjusted for age, gender, race, education level, family income level, BMI, smoking status, alcohol intake, diabetes and hypertension.
