# Supplementary material for: The serum uric acid-to-high-density lipoprotein cholesterol ratio is a predictor for all-cause and cardiovascular disease mortality: a cross-sectional study
Source: Front Endocrinol (Lausanne). 2024 Sep 13;15:1417485. doi: 10.3389/fendo.2024.1417485 (PMC11427315; doi:10.3389/fendo.2024.1417485)
Supplement: Supplementary file 2 [file DataSheet2.pdf]

Figure legends:

Figure S1: Dose-response associations of UHR with all-cause and CVD mortality in different populations.

Association between UHR and all-cause and CVD mortality in patients with diabetes (A-B), obesity (C-D) and CVD (E-F); The associations were examined by multivariable Cox regression models with restricted cubic splines. HRs adjusted for age, gender, race, education level, family income level, BMI, smoking status, alcohol intake, diabetes and hypertension except the corresponding stratification variable. Solid lines represent estimates of HRs and dashed lines represent 95% CIs.

Figure S2: Forest plot of stratified analyses of UHA and all-cause mortality in patients with diabetes.

HRs adjusted for age, gender, race, education level, family income level, BMI, smoking status, alcohol intake, diabetes and hypertension except the corresponding stratification variable.

Figure S3: Forest plot of stratified analyses of UHA and CVD mortality in patients with diabetes.

HRs adjusted for age, gender, race, education level, family income level, BMI, smoking status, alcohol intake, diabetes and hypertension except the corresponding stratification variable.

Figure S4: Forest plot of stratified analyses of UHA and all-cause mortality in patients with diabetes.

HRs adjusted for age, gender, race, education level, family income level, BMI, smoking status, alcohol intake, diabetes and hypertension except the corresponding stratification variable.

Figure S5: Forest plot of stratified analyses of UHA and CVD mortality in patients with diabetes.

HRs adjusted for age, gender, race, education level, family income level, BMI, smoking status, alcohol intake, diabetes and hypertension except the corresponding stratification variable.

Figure S6: Forest plot of stratified analyses of UHA and all-cause mortality in patients with CVD.

HRs adjusted for age, gender, race, education level, family income level, BMI, smoking status, alcohol intake, diabetes and hypertension except the corresponding stratification variable.

Figure S7: Forest plot of stratified analyses of UHA and CVD mortality in patients with CVD.

HRs adjusted for age, gender, race, education level, family income level, BMI, smoking status, alcohol intake, diabetes and hypertension except the corresponding stratification variable.
