# Supplementary figures and images for: The serum uric acid-to-high-density lipoprotein cholesterol ratio is a predictor for all-cause and cardiovascular disease mortality: a cross-sectional study
Source: Front Endocrinol (Lausanne). 2024 Sep 13;15:1417485. doi: 10.3389/fendo.2024.1417485 (PMC11427315; doi:10.3389/fendo.2024.1417485)

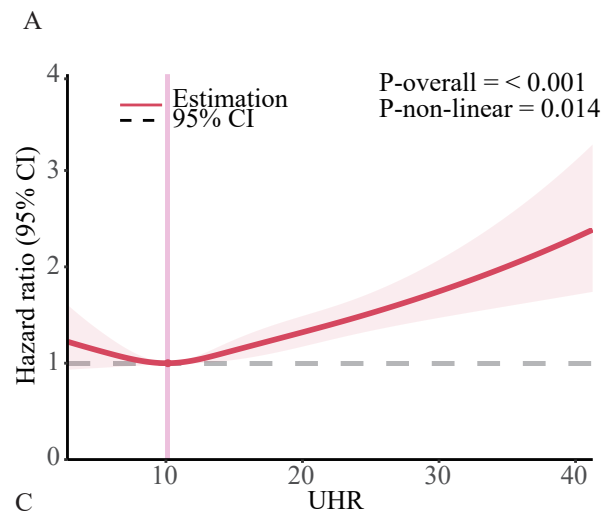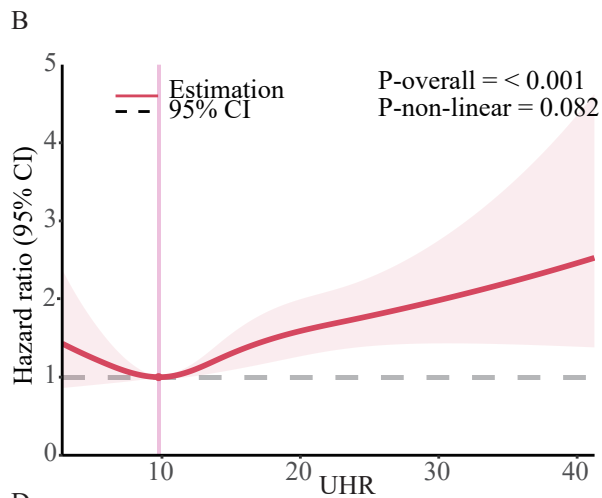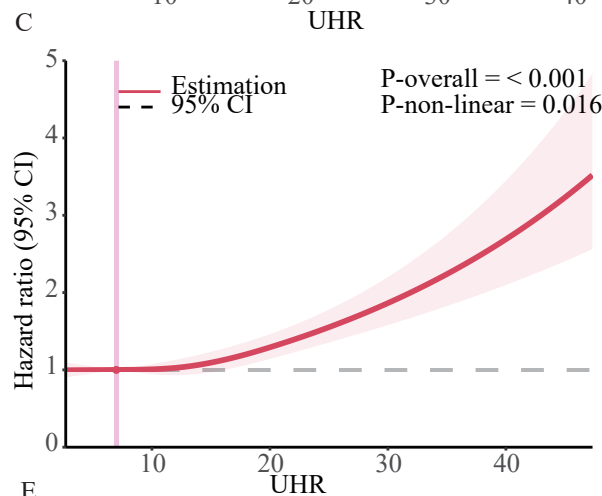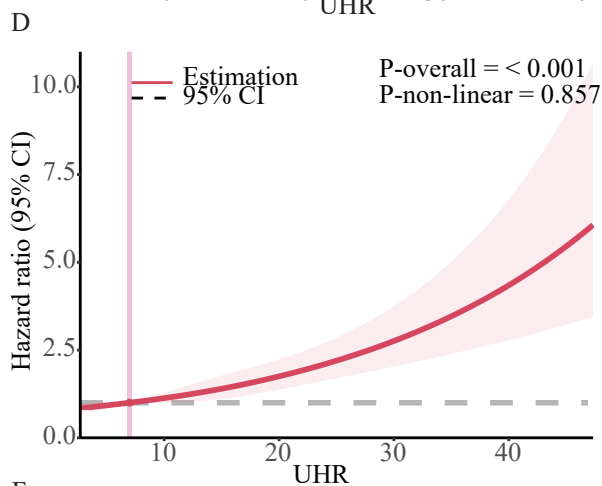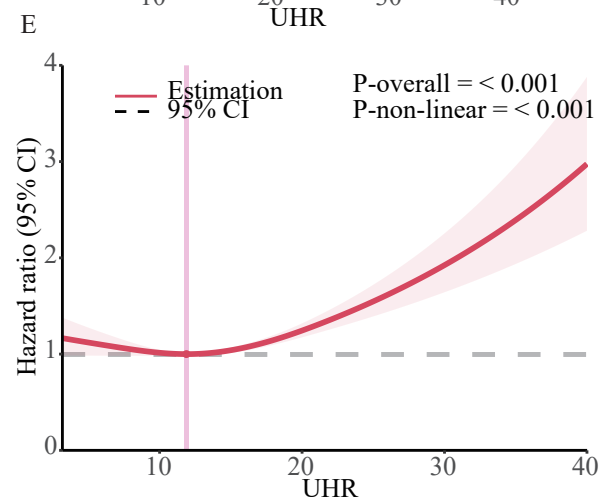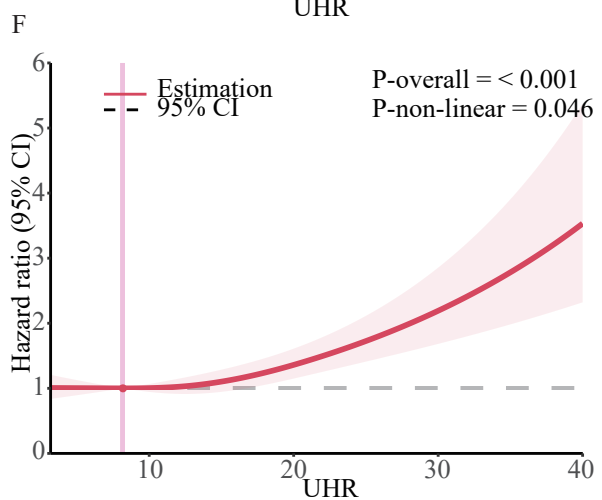

Supplement: Supplementary file 3 [file DataSheet3.pdf]
