# Supplementary material for: The serum uric acid-to-high-density lipoprotein cholesterol ratio is a predictor for all-cause and cardiovascular disease mortality: a cross-sectional study
Source: Front Endocrinol (Lausanne). 2024 Sep 13;15:1417485. doi: 10.3389/fendo.2024.1417485 (PMC11427315; doi:10.3389/fendo.2024.1417485)

Variable	Count	Percent	HR (95% CI)	*P* value	*P* for interaction
Overall	7796	100	1.33 (1.18 to 1.51)	<0.001	
sex					0.007
Male	4048	51.9	1.16 (0.98 to 1.36)	0.082	
Female	3748	48.1	1.63 (1.34 to 1.98)	<0.001	
Age					0.18
<30	165	2.1	6.71 (0.62 to 72.56)	0.117	
30-40	416	5.3	0.60 (0.25 to 1.46)	0.262	
40-50	944	12.1	1.30 (0.71 to 2.40)	0.395	
≥50	6271	80.4	1.40 (1.23 to 1.59)	<0.001	
Race					0.695
Mexican American	1602	20.5	1.49 (1.08 to 2.06)	0.016	
Non–Hispanic White	2780	35.7	1.28 (1.10 to 1.50)	0.002	
Non-Hispanic Black	1946	25	1.21 (0.96 to 1.51)	0.103	
Other Race	1468	18.8	1.43 (1.00 to 2.04)	0.047	
BMI					0.714
<25	1075	13.8	1.46 (1.13 to 1.89)	0.003	
25-30	2329	29.9	1.56 (1.27 to 1.92)	<0.001	
≥30	4392	56.3	1.41 (1.21 to 1.65)	<0.001	
Education					0.233
Less than high school	2905	37.3	1.35 (1.16 to 1.59)	<0.001	
High school	1761	22.6	1.28 (1.00 to 1.63)	0.051	
College or above	3118	40	1.40 (1.15 to 1.71)	0.001	
Missing data	12	0.2			
Family income level					0.402
<1.30	2514	32.2	1.23 (1.00 to 1.50)	0.048	
1.31-3.50	2840	36.4	1.46 (1.24 to 1.72)	<0.001	
≥3.50	1684	21.6	1.37 (1.02 to 1.84)	0.036	
Missing data	758	9.7	1.14 (0.76 to 1.71)	0.528	
Hypertension					0.864
No	1596	20.5	1.32 (0.98 to 1.78)	0.065	
Yes	6198	79.5	1.28 (1.12 to 1.46)	<0.001	
CVD					0.354
No	5819	74.7	1.19 (1.02 to 1.40)	0.031	
Yes	1975	25.3	1.34 (1.12 to 1.60)	0.001	
Alcohol intake					0.088
Heavy drinking	569	7.3	1.02 (0.65 to 1.61)	0.924	
Moderate drinking	457	5.9	1.50 (0.93 to 2.43)	0.098	
Non drinkers	6257	80.3	1.33 (1.15 to 1.54)	<0.001	
Missing data	513	6.6	1.93 (1.27 to 2.94)	0.002	
Smoking status					0.172
Current smokers	1275		0.97 (0.73 to 1.29)	0.847	
Former smokers	2659		1.37 (1.15 to 1.63)	<0.001	
Non smokers	3854		1.40 (1.16 to 1.70)	0.001	

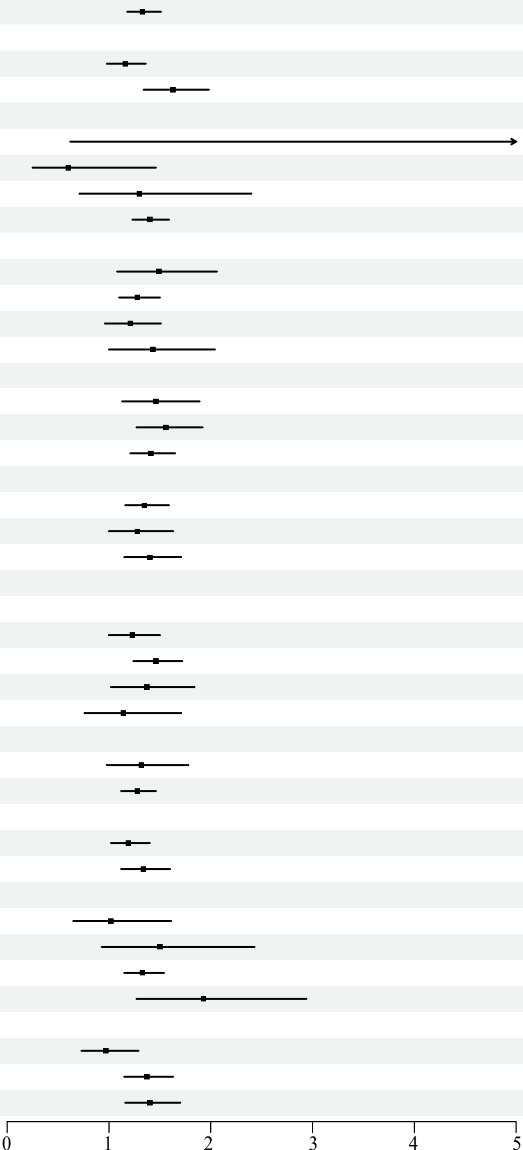

Supplement: Supplementary file 4 [file DataSheet4.pdf]
