# Supplementary material for: The serum uric acid-to-high-density lipoprotein cholesterol ratio is a predictor for all-cause and cardiovascular disease mortality: a cross-sectional study
Source: Front Endocrinol (Lausanne). 2024 Sep 13;15:1417485. doi: 10.3389/fendo.2024.1417485 (PMC11427315; doi:10.3389/fendo.2024.1417485)

Variable	Count	Percent	HR (95% CI)	*P* value	*P* for interaction
Overall	7796	100	1.63 (1.29 to 2.06)	<0.001	
sex					0.841
Male	4048	51.9	1.58 (1.20 to 2.09)	0.001	
Female	3748	48.1	1.65 (1.14 to 2.39)	0.008	
Age					<0.001
<30	165	2.1			
30-40	416	5.3	1.16 (0.18 to 7.32)	0.873	
40-50	944	12.1	1.42 (0.48 to 4.20)	0.528	
≥50	6271	80.4	1.74 (1.37 to 2.20)	<0.001	
Race					0.694
Mexican American	1602	20.5	1.75 (0.85 to 3.62)	0.129	
Non–Hispanic White	2780	35.7	1.60 (1.18 to 2.17)	0.002	
Non-Hispanic Black	1946	25	1.25 (0.80 to 1.93)	0.324	
Other Race	1468	18.8	1.79 (0.80 to 4.00)	0.159	
BMI					0.472
<25	1075	13.8	1.19 (0.63 to 2.22)	0.592	
25-30	2329	29.9	1.88 (1.25 to 2.84)	0.002	
≥30	4392	56.3	1.86 (1.38 to 2.52)	<0.001	
Education					<0.001
Less than high school	2905	37.3	1.75 (1.24 to 2.47)	0.002	
High school	1761	22.6	1.98 (1.28 to 3.05)	0.002	
College or above	3118	40	1.32 (0.87 to 2.01)	0.194	
Missing data	12	0.2			
Family income level					0.142
<1.30	2514	32.2	1.13 (0.71 to 1.80)	0.601	
1.31-3.50	2840	36.4	1.68 (1.25 to 2.26)	0.001	
≥3.50	1684	21.6	2.20 (1.40 to 3.47)	0.001	
Missing data	758	9.7	2.02 (0.95 to 4.29)	0.067	
Hypertension					0.286
No	1596	20.5	2.11 (1.21 to 3.66)	0.008	
Yes	6198	79.5	1.50 (1.15 to 1.94)	0.003	
CVD					0.566
No	5819	74.7	1.38 (0.98 to 1.93)	0.063	
Yes	1975	25.3	1.54 (1.17 to 2.04)	0.002	
Alcohol intake					0.62
Heavy drinking	569	7.3	1.70 (0.77 to 3.76)	0.189	
Moderate drinking	457	5.9	1.86 (0.73 to 4.76)	0.193	
Non drinkers	6257	80.3	1.66 (1.28 to 2.15)	<0.001	
Missing data	513	6.6	0.82 (0.34 to 1.98)	0.665	
Smoking status					0.017
Current smokers	1275		0.86 (0.52 to 1.41)	0.554	
Former smokers	2659		2.15 (1.55 to 3.00)	<0.001	
Non smokers	3854		1.51 (1.07 to 2.14)	0.02	

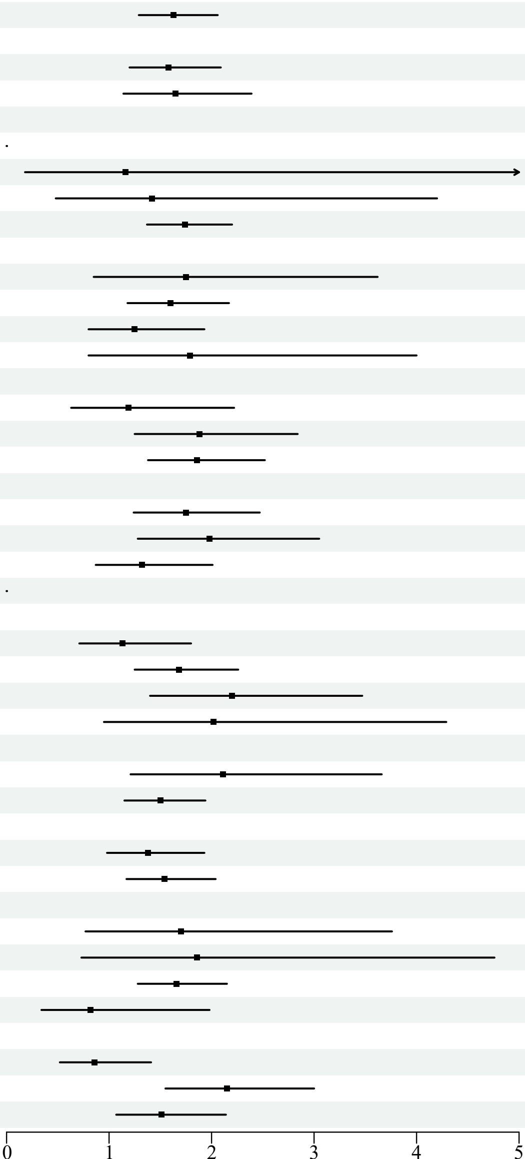

Supplement: Supplementary file 5 [file DataSheet5.pdf]
