# Supplementary material for: The serum uric acid-to-high-density lipoprotein cholesterol ratio is a predictor for all-cause and cardiovascular disease mortality: a cross-sectional study
Source: Front Endocrinol (Lausanne). 2024 Sep 13;15:1417485. doi: 10.3389/fendo.2024.1417485 (PMC11427315; doi:10.3389/fendo.2024.1417485)

Variable	Count	Percent	HR (95% CI)	*P* value	*P* for interaction
Overall	17547	100	1.55 (1.27 to 1.89)	<0.001	
sex					<0.001
Male	7615	43.4	0.82 (0.58 to 1.16)	0.263	
Female	9932	56.6	2.52 (2.01 to 3.17)	<0.001	
Age					0.984
<30	2466	14.1	2.50 (0.26 to 24.25)	0.43	
30-40	3026	17.2	2.01 (0.67 to 6.01)	0.213	
40-50	3227	18.4	1.84 (0.85 to 3.98)	0.123	
≥50	8828	50.3	1.74 (1.40 to 2.17)	<0.001	
Race					0.941
Mexican American	3445	19.6	1.65 (0.88 to 3.12)	0.12	
Non–Hispanic White	7268	41.4	1.53 (1.19 to 1.98)	0.001	
Non-Hispanic Black	4435	25.3	1.40 (0.98 to 2.00)	0.066	
Other Race	2399	13.7	1.65 (0.70 to 3.91)	0.251	
Education					<0.001
Less than high school	4848	27.6	1.73 (1.22 to 2.44)	0.002	
High school	4269	24.3	1.51 (1.02 to 2.23)	0.041	
College or above	8416	48	1.45 (1.05 to 1.99)	0.024	
Missing data	14	0.1			
Family income level					0.436
<1.30	5181	29.5	1.86 (1.32 to 2.63)	<0.001	
1.31-3.50	6306	35.9	1.38 (1.03 to 1.84)	0.031	
≥3.50	4582	26.1	1.75 (1.09 to 2.82)	0.02	
Missing data	1478	8.4	1.19 (0.69 to 2.03)	0.535	
Diabetes					0.005
No	13155	75	1.23 (0.96 to 1.58)	0.096	
Yes	4392	25	2.02 (1.53 to 2.68)	<0.001	
Hypertension					0.143
No	6249	35.6	2.11 (1.23 to 3.62)	0.007	
Yes	11297	64.4	1.35 (1.08 to 1.70)	0.01	
CVD					0.833
No	15285	87.1	1.42 (1.08 to 1.87)	0.013	
Yes	2260	12.9	1.49 (1.18 to 1.88)	0.001	
Alcohol intake					0.222
Heavy drinking	1983	11.3	0.88 (0.44 to 1.78)	0.722	
Moderate drinking	1094	6.2	1.05 (0.52 to 2.16)	0.884	
Non drinkers	13605	77.5	1.75 (1.37 to 2.22)	<0.001	
Missing data	865	4.9	1.11 (0.45 to 2.71)	0.819	
Smoking status					0.111
Current smokers	3166		1.01 (0.61 to 1.65)	0.983	
Former smokers	4685		1.95 (1.46 to 2.63)	<0.001	
Non smokers	9683		1.45 (1.07 to 1.96)	0.017	

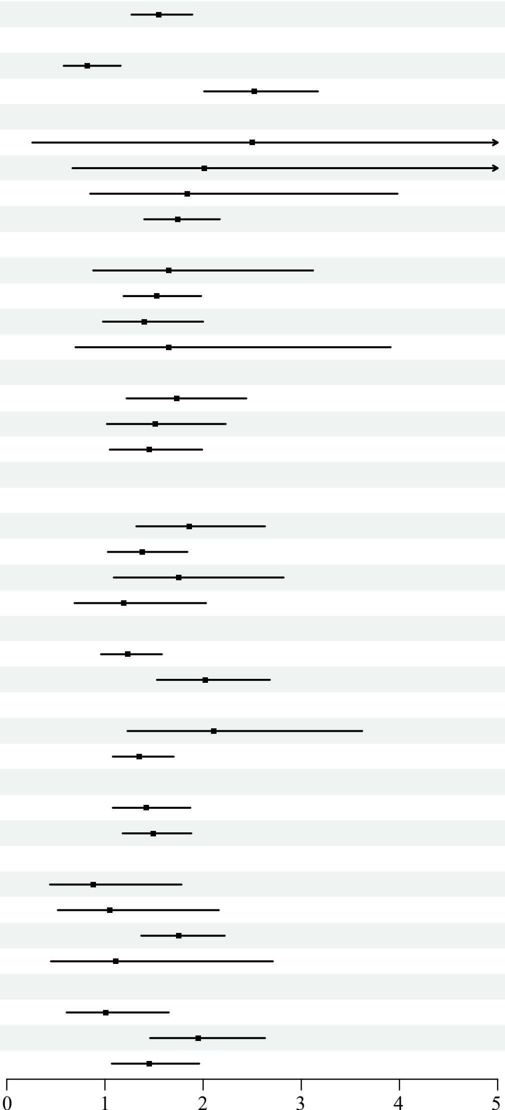

Supplement: Supplementary file 7 [file DataSheet7.pdf]
