# Supplementary material for: The serum uric acid-to-high-density lipoprotein cholesterol ratio is a predictor for all-cause and cardiovascular disease mortality: a cross-sectional study
Source: Front Endocrinol (Lausanne). 2024 Sep 13;15:1417485. doi: 10.3389/fendo.2024.1417485 (PMC11427315; doi:10.3389/fendo.2024.1417485)

| Variable | Count | Percent | HR (95% CI) | *P* value | *P* for interaction |
|---|---|---|---|---|---|
| Overall | 5191 | 100 | 1.31 (1.09 to 1.59) | 0.005 | |
| sex | | | | | 0.001 |
| Male | 2938 | 56.6 | 1.16 (0.93 to 1.44) | 0.181 | |
| Female | 2253 | 43.4 | 2.02 (1.53 to 2.66) | <0.001 | |
| Age | | | | | 0.803 |
| <30 | 62 | 1.2 | 5.97 (0.28 to 124.99) | 0.25 | |
| 30-40 | 146 | 2.8 | 1.41 (0.30 to 6.68) | 0.661 | |
| 40-50 | 370 | 7.1 | 0.89 (0.32 to 2.47) | 0.83 | |
| ≥50 | 4613 | 88.9 | 1.29 (1.05 to 1.57) | 0.013 | |
| Race | | | | | 0.376 |
| Mexican American | 602 | 11.6 | 2.11 (1.16 to 3.84) | 0.015 | |
| Non–Hispanic White | 2911 | 56.1 | 1.26 (1.00 to 1.58) | 0.05 | |
| Non-Hispanic Black | 1062 | 20.5 | 1.57 (1.13 to 2.18) | 0.007 | |
| Other Race | 616 | 11.9 | 1.35 (0.67 to 2.73) | 0.399 | |
| BMI | | | | | 0.267 |
| <25 | 1176 | 22.7 | 2.05 (1.24 to 3.37) | 0.005 | |
| 25-30 | 1755 | 33.8 | 1.44 (1.04 to 1.99) | 0.028 | |
| ≥30 | 2260 | 43.5 | 1.29 (1.00 to 1.66) | 0.049 | |
| Education | | | | | <0.001 |
| Less than high school | 1848 | 35.6 | 1.55 (1.18 to 2.04) | 0.002 | |
| High school | 1274 | 24.5 | 0.98 (0.67 to 1.42) | 0.909 | |
| College or above | 2057 | 39.6 | 1.46 (1.12 to 1.90) | 0.005 | |
| Missing data | 12 | 0.2 | | | |
| Family income level | | | | | 0.389 |
| <1.30 | 1765 | 34 | 1.50 (1.12 to 2.02) | 0.006 | |
| 1.31-3.50 | 1922 | 37 | 1.38 (1.04 to 1.84) | 0.025 | |
| ≥3.50 | 1057 | 20.4 | 0.97 (0.62 to 1.51) | 0.886 | |
| Missing data | 447 | 8.6 | 1.22 (0.80 to 1.84) | 0.352 | |
| Diabetes | | | | | 0.195 |
| No | 3216 | 62 | 1.15 (0.89 to 1.47) | 0.282 | |
| Yes | 1975 | 38 | 1.43 (1.11 to 1.84) | 0.005 | |
| Hypertension | | | | | 0.392 |
| No | 874 | 16.8 | 1.55 (0.96 to 2.48) | 0.071 | |
| Yes | 4316 | 83.2 | 1.25 (1.02 to 1.54) | 0.03 | |
| Alcohol intake | | | | | 0.238 |
| Heavy drinking | 437 | 8.4 | 0.55 (0.21 to 1.40) | 0.207 | |
| Moderate drinking | 385 | 7.4 | 1.03 (0.51 to 2.07) | 0.936 | |
| Non drinkers | 4085 | 78.7 | 1.38 (1.13 to 1.69) | 0.001 | |
| Missing data | 284 | 5.5 | 1.38 (0.58 to 3.30) | 0.465 | |
| Smoking status | | | | | 0.418 |
| Current smokers | 1047 | 20.2 | 1.04 (0.68 to 1.59) | 0.849 | |
| Former smokers | 2124 | 40.9 | 1.30 (0.98 to 1.72) | 0.072 | |
| Non smokers | 2019 | 38.9 | 1.44 (1.09 to 1.92) | 0.012 | |

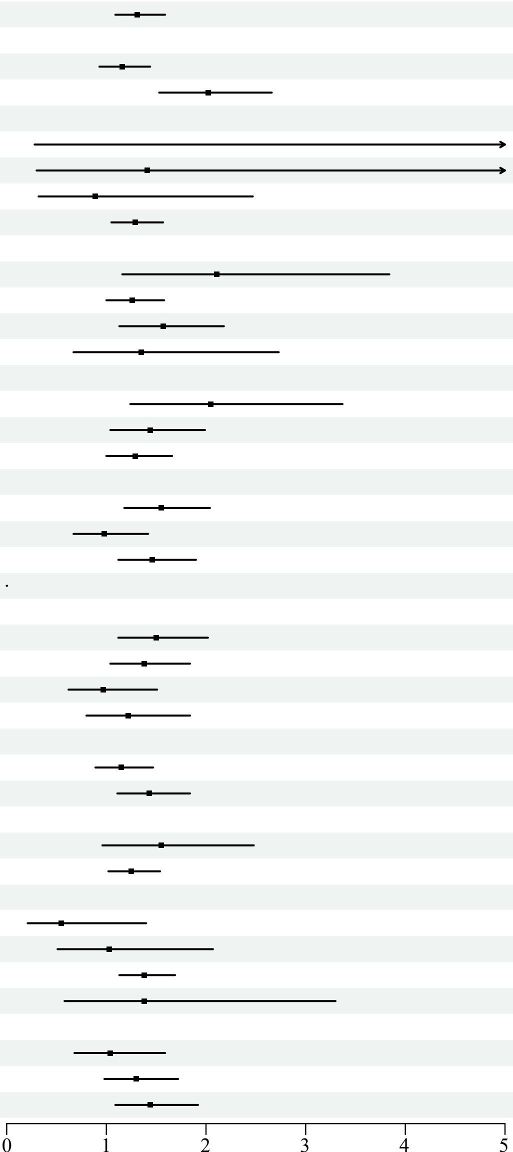

Supplement: Supplementary file 8 [file DataSheet8.pdf]
