# Supplementary material for: The serum uric acid-to-high-density lipoprotein cholesterol ratio is a predictor for all-cause and cardiovascular disease mortality: a cross-sectional study
Source: Front Endocrinol (Lausanne). 2024 Sep 13;15:1417485. doi: 10.3389/fendo.2024.1417485 (PMC11427315; doi:10.3389/fendo.2024.1417485)

| Variable | Count | Percent | HR (95% CI) | *P* value | *P* for interaction |
|---|---|---|---|---|---|
| Overall | 5191 | 100 | 1.46 (1.21 to 1.76) | <0.001 | |
| sex | | | | | 0.989 |
| Male | 2938 | 56.6 | 1.47 (1.18 to 1.83) | 0.001 | |
| Female | 2253 | 43.4 | 1.47 (0.99 to 2.18) | 0.058 | |
| Age | | | | | 0.276 |
| <30 | 62 | 1.2 | | | |
| 30-40 | 146 | 2.8 | 6.88 (1.11 to 42.48) | 0.038 | |
| 40-50 | 370 | 7.1 | 0.64 (0.18 to 2.25) | 0.489 | |
| ≥50 | 4613 | 4613 | 1.43 (1.17 to 1.76) | 0.001 | |
| Race | | | | | 0.111 |
| Mexican American | 602 | 11.6 | 2.67 (1.21 to 5.92) | 0.015 | |
| Non–Hispanic White | 2911 | 56.1 | 1.43 (1.14 to 1.79) | 0.002 | |
| Non-Hispanic Black | 1062 | 20.5 | 0.95 (0.57 to 1.60) | 0.849 | |
| Other Race | 616 | 11.9 | 2.01 (0.85 to 4.76) | 0.113 | |
| BMI | | | | | 0.687 |
| <25 | 1176 | 22.7 | 1.61 (0.88 to 2.95) | 0.125 | |
| 25-30 | 1755 | 33.8 | 1.73 (1.23 to 2.42) | 0.002 | |
| ≥30 | 2260 | 43.5 | 1.45 (1.09 to 1.94) | 0.012 | |
| Education | | | | | <0.001 |
| Less than high school | 1848 | 35.6 | 1.61 (1.16 to 2.25) | 0.005 | |
| High school | 1274 | 24.5 | 1.18 (0.79 to 1.78) | 0.423 | |
| College or above | 2057 | 39.6 | 1.50 (1.12 to 2.02) | 0.007 | |
| Missing data | 12 | 0.2 | | | |
| Family income level | | | | | 0.431 |
| <1.30 | 1765 | 34 | 1.35 (0.97 to 1.88) | 0.078 | |
| 1.31-3.50 | 1922 | 37 | 1.37 (1.02 to 1.84) | 0.038 | |
| ≥3.50 | 1057 | 20.4 | 1.98 (1.30 to 3.01) | 0.001 | |
| Missing data | 447 | 8.6 | 1.11 (0.55 to 2.25) | 0.766 | |
| Diabetes | | | | | 0.443 |
| No | 3216 | 62 | 1.30 (1.00 to 1.67) | 0.049 | |
| Yes | 1975 | 38 | 1.52 (1.16 to 2.00) | 0.002 | |
| Hypertension | | | | | 0.45 |
| No | 874 | 16.8 | 1.76 (1.04 to 2.98) | 0.036 | |
| Yes | 4316 | 83.2 | 1.39 (1.11 to 1.73) | 0.003 | |
| Alcohol intake | | | | | 0.463 |
| Heavy drinking | 437 | 8.4 | 1.87 (0.89 to 3.94) | 0.101 | |
| Moderate drinking | 385 | 7.4 | 1.26 (0.67 to 2.39) | 0.471 | |
| Non drinkers | 4085 | 78.7 | 1.49 (1.19 to 1.88) | 0.001 | |
| Missing data | 284 | 5.5 | 0.76 (0.30 to 1.96) | 0.575 | |
| Smoking status | | | | | 0.194 |
| Current smokers | 1047 | 20.2 | 1.06 (0.61 to 1.83) | 0.836 | |
| Former smokers | 2124 | 40.9 | 1.79 (1.36 to 2.35) | <0.001 | |
| Non smokers | 2019 | 38.9 | 1.27 (0.90 to 1.78) | 0.174 | |

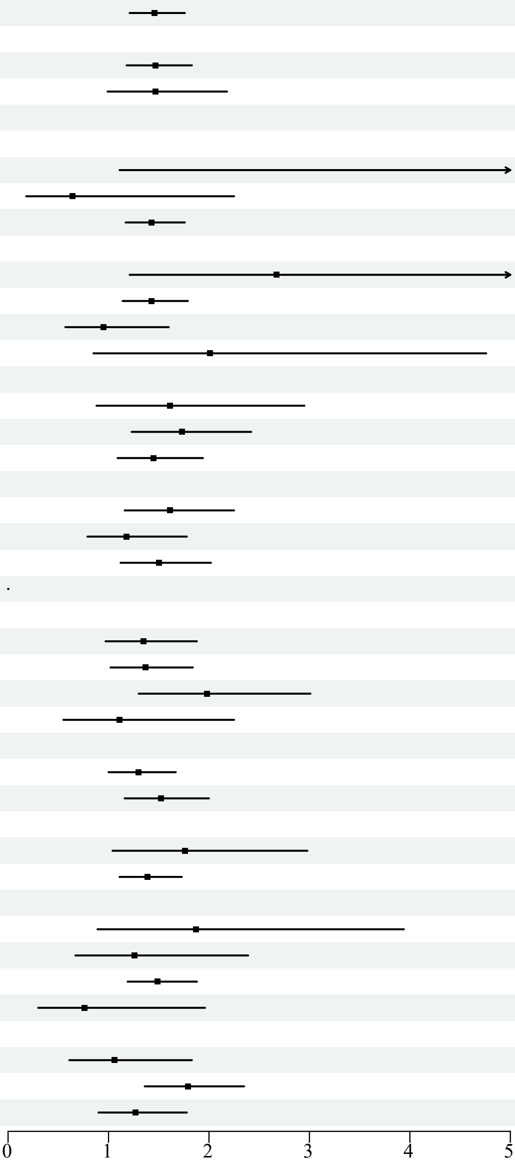

Supplement: Supplementary file 9 [file DataSheet9.pdf]
