# Supplementary material for: The serum uric acid-to-high-density lipoprotein cholesterol ratio is a predictor for all-cause and cardiovascular disease mortality: a cross-sectional study
Source: Front Endocrinol (Lausanne). 2024 Sep 13;15:1417485. doi: 10.3389/fendo.2024.1417485 (PMC11427315; doi:10.3389/fendo.2024.1417485)

Variable	Count	Percent	HR (95% CI)	*P* value	*P* for interaction
Overall	17547	100	1.28 (1.16 to 1.42)	<0.001	
sex					<0.001
Male	7615	43.4	0.75 (0.63 to 0.89)	0.001	
Female	9932	56.6	1.89 (1.65 to 2.16)	<0.001	
Age					0.623
<30	2466	14.1	0.86 (0.38 to 1.95)	0.716	
30-40	3026	17.2	1.50 (0.86 to 2.61)	0.149	
40-50	3227	18.4	1.53 (0.96 to 2.44)	0.073	
≥50	8828	50.3	1.45 (1.30 to 1.62)	<0.001	
Race					0.131
Mexican American	3445	19.6	1.19 (0.89 to 1.59)	0.251	
Non–Hispanic White	7268	41.4	1.26 (1.12 to 1.42)	<0.001	
Non-Hispanic Black	4435	25.3	1.50 (1.23 to 1.84)	<0.001	
Other Race	2399	13.7	0.89 (0.58 to 1.35)	0.572	
Education					<0.001
Less than high school	4848	27.6	1.43 (1.23 to 1.66)	<0.001	
High school	4269	24.3	1.17 (0.96 to 1.43)	0.129	
College or above	8416	48	1.26 (1.06 to 1.50)	0.007	
Missing data	14	0.1			
Family income level					0.923
<1.30	5181	29.5	1.35 (1.13 to 1.63)	0.001	
1.31-3.50	6306	35.9	1.26 (1.09 to 1.47)	0.002	
≥3.50	4582	26.1	1.31 (1.05 to 1.63)	0.016	
Missing data	1478	8.4	1.20 (0.87 to 1.66)	0.27	
Diabetes					0.128
No	13155	75	1.16 (1.01 to 1.34)	0.04	
Yes	4392	25	1.38 (1.18 to 1.61)	<0.001	
Hypertension					0.151
No	6249	35.6	1.46 (1.11 to 1.92)	0.007	
Yes	11297	64.4	1.16 (1.02 to 1.31)	0.02	
CVD					0.43
No	15285	87.1	1.18 (1.02 to 1.36)	0.022	
Yes	2260	12.9	1.30 (1.10 to 1.53)	0.002	
Alcohol intake					0.136
Heavy drinking	1983	11.3	0.92 (0.62 to 1.36)	0.673	
Moderate drinking	1094	6.2	0.97 (0.65 to 1.43)	0.862	
Non drinkers	13605	77.5	1.39 (1.23 to 1.58)	<0.001	
Missing data	865	4.9	1.14 (0.74 to 1.75)	0.55	
Smoking status					<0.001
Current smokers	3166	18	1.00 (0.78 to 1.29)	0.984	
Former smokers	4685	26.7	1.47 (1.26 to 1.71)	<0.001	
Non smokers	9683	55.2	1.18 (1.01 to 1.38)	0.042	
Missing data	13	0.1			

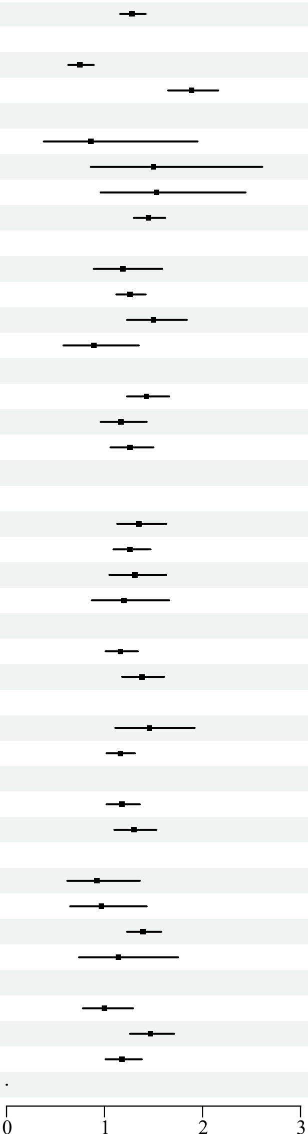

Supplement: Supplementary file 6 [file DataSheet6.pdf]
